# Characterization of Cytochrome P450s with Key Roles in Determining Herbicide Selectivity in Maize

**DOI:** 10.1021/acsomega.2c01705

**Published:** 2022-05-11

**Authors:** Melissa Brazier-Hicks, Sara Franco-Ortega, Philip Watson, Blandine Rougemont, Jonathan Cohn, Richard Dale, Tim R. Hawkes, Alina Goldberg-Cavalleri, Nawaporn Onkokesung, Robert Edwards

**Affiliations:** † Agriculture, School of Natural and Environmental Sciences, Newcastle University, Newcastle upon Tyne NE1 7RU, U.K.; ‡ Syngenta, Jealott’s Hill, Bracknell, Berkshire RG42 6EY, U.K.; § Syngenta Crop Protection, LLC, 9 Davis Drive, Research Triangle Park, Durham, North Carolina 27709-2257, United States

## Abstract

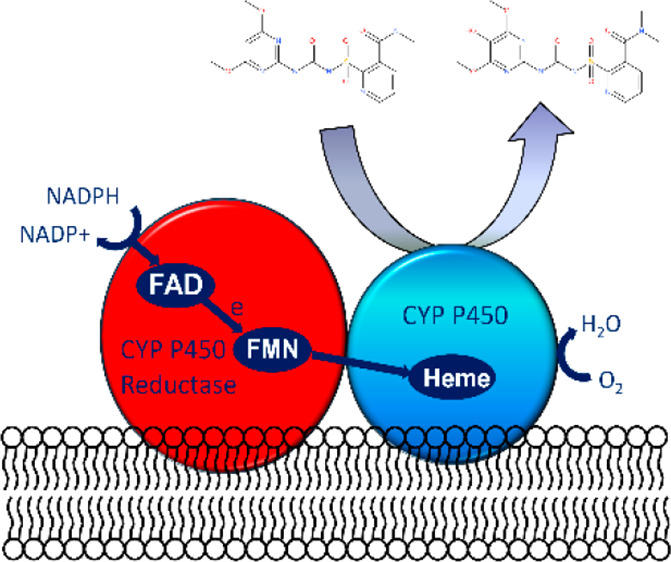

Safeners such as metcamifen and benoxacor are widely used in maize to enhance the selectivity of herbicides through the induction of key detoxifying enzymes, notably cytochrome P450 monooxygenases (CYPs). Using a combination of transcriptomics, proteomics, and functional assays, the safener-inducible CYPs responsible for herbicide metabolism in this globally important crop have been identified. A total of 18 CYPs belonging to clans 71, 72, 74, and 86 were safener-induced, with the respective enzymes expressed in yeast and screened for activity toward thiadiazine (bentazon), sulfonylurea (nicosulfuron), and triketone (mesotrione and tembotrione) chemistries. Herbicide metabolism was largely restricted to family CYP81A members from clan 71, notably CYP81A9, CYP81A16, and CYP81A2. Quantitative transcriptomics and proteomics showed that CYP81A9/CYP81A16 were dominant enzymes in safener-treated field maize, whereas only CYP81A9 was determined in sweet corn. The relationship between CYP81A sequence and activities were investigated by splicing CYP81A2 and CP81A9 together as a series of recombinant chimeras. CYP81A9 showed wide ranging activities toward the three herbicide chemistries, while CYP81A2 uniquely hydroxylated bentazon in multiple positions. The plasticity in substrate specificity of CYP81A9 toward multiple herbicides resided in the second quartile of its N terminal half. Further phylogenetic analysis of CYP81A9 showed that the maize enzyme was related to other CYP81As linked to agrochemical metabolism in cereals and wild grasses, suggesting this clan 71 CYP has a unique function in determining herbicide selectivity in arable crops.

## Introduction

**Figure 1:**
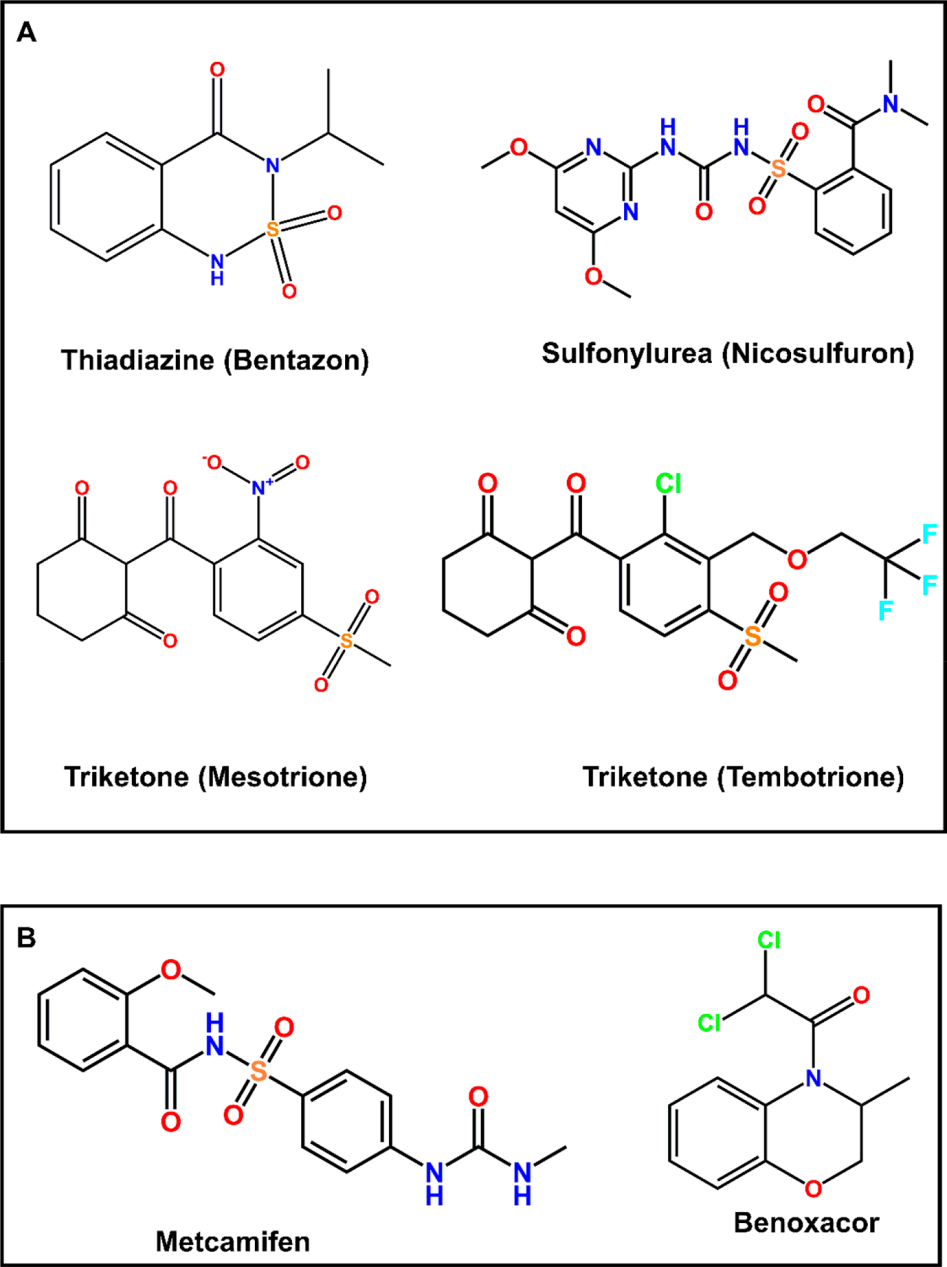
Chemical structures of (A) herbicides (bentazon, nicosulfuron, mesotrione, tembotrione) and (B) safeners (benoxacor, metcamifen), used in the primary characterization of the CYPs in this study.

Selective herbicides are a fundamental tool underpinning modern arable agriculture, allowing weeds to be controlled without damaging the crop.^1,2^ As the proteins targeted by herbicides in crops and weeds are typically conserved and similarly sensitive to inhibition, selectivity is determined through controlling the bioavailability of the applied chemistry. This is typically achieved through differential metabolism, with the herbicide being more rapidly detoxified in the crop than in the competing weed by a complex of enzymes and transporters termed the “xenome”.^2,3^ In some cases, the crop xenome is inherently more active than that of the weed, such that herbicide selectivity is exhibited constitutively.^4,5^ However, selectivity is often dependent on the use of safeners, compounds which on pre- or coapplication with the herbicide selectively enhance the activity of the xenome in the crops.^1,6,7^ This enhancement is associated with the coordinated induction of xenome genes, notably cytochrome P450 monooxygenases (CYPs), glutathione transferases (GSTs) and ATP-binding cassette (ABC) transporters.^2,6,8^ As a globally important crop, weed control in maize (*Zea mays* L.) is of critical importance in ensuring yield, with many classes of selective herbicides developed. These compounds represent different chemical classes with notable examples being a diverse range of triketones and sulfonylureas and the thiadiazine bentazon (Figure 1), extending to benzoylpyrazoles, sulfonanilides, imidazolinones, and phenylureas (Figure S1). In maize, a primary mechanism of detoxification of all these herbicides is CYP-mediated metabolism by hydroxylation and dealkylation.^9^

CYPs (E.C. 1.14.) are a superfamily of membrane bound oxidoreductases with multiple roles in primary, secondary, and xenobiotic metabolism.^9−11^ In plants, CYPs are grouped into 10 clans, with 4 of these composed of multiple CYP families. The respective gene families are often large, with maize CYPs comprising 263 coding sequences and 7 pseudogenes.^12^ While the available evidence suggests the diversity of CYPs in crops and weeds is not fundamentally different,^13^ the expression of these enzymes is normally greater in domesticated species such as maize, especially when associated with safener use.^14,15^ However, little is known as to the functional activity of the maize CYP superfamily, posing the question as to how many of these enzymes are involved in the metabolism of these diverse herbicide chemistries and how they are regulated by safeners.

Understanding the range of CYPs involved in detoxifying herbicides in a major cereal crop such as maize and their responsiveness to safeners is now critical to understanding their importance in selectivity for both current and future chemical weed control agents. The objective of the study is to define the “CYPome” responsible for conferring herbicide selectivity in maize. CYPs responsible for herbicide detoxification in maize have been targeted for characterization using safeners to help identify members of the enzyme class responsible for providing protection against chemical injury. To confirm enhanced CYP-based metabolism, maize seedlings have been treated with the triketone herbicide mesotrione, following exposure to two different safener chemistries, benoxacor and metcamifen (Figure 1). The effect of the safeners on mesotrione metabolism was then monitored over time. While benoxacor has been used to safen a range of herbicides in maize,^16^ the recently released aromatic sulfonamide (metcamifen) has broader ranging safening activities in a range of cereals.^8^ Intriguingly, recent studies have shown that different sulfonamide safeners invoke different inductions of xenome genes in other cereal crops such as rice and wheat.^7,8^ As such, it was of interest to use two different safener chemistries to potentially induce distinct xenome responses including the differential expression of the maize CYPs. Using this strategy, we have used global transcriptomics to identify both constitutively expressed and safener-responsive CYPs. These candidates were cloned and functionally characterized in recombinant yeast by testing their activity toward key herbicides. In addition, the effect of safener treatment on the expression of these CYPs has been quantified in maize tissues and cell cultures at the transcript and proteome levels respectively, to help define their importance in herbicide metabolism *in planta*.

## Results

### Herbicide Safeners Selectively Enhance CYP-Mediated Detoxification of [^14^C]-Mesotrione in Maize

**Figure 2:**
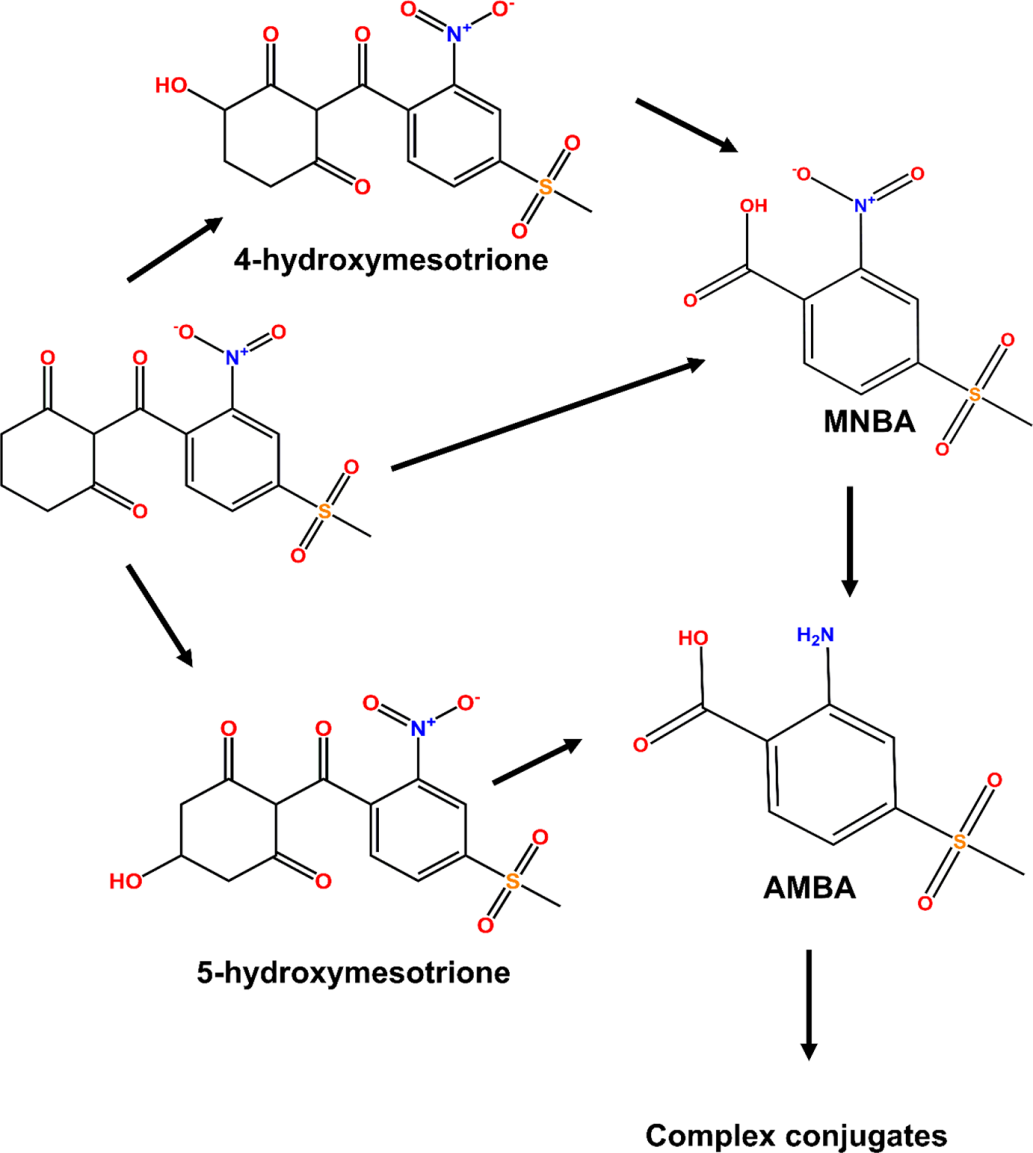
Reaction scheme of CYP-mediated biotransformation of mesotrione in maize. MNBA: 4-methylsulfonyl-2-nitrobenzoic acid, AMBA: 2-amino-4-methyl sulfonyl benzoic acid.

To evaluate the effect of safeners on the CYP-mediated metabolism of a herbicide, maize seedlings were transiently exposed to either metcamifen or benoxacor (Figure 1) and then fed via the roots with the radiolabeled triketone [^14^C]-mesotrione (Figure S2). Mesotrione was selected, as it can undergo three distinct CYP-mediated detoxification reactions, namely 4-hydroxylation, 5-hydroxylation, and oxidative cleavage of the herbicide to form the metabolite 4-methylsulfonyl-2-nitrobenzoic acid (MNBA; Figure 2).^29^ The identity of the CYPs responsible for these three distinct biotransformations has not been reported, so it was of interest to determine whether the respective activities were differentially sensitive to safener action.

**Figure 3:**
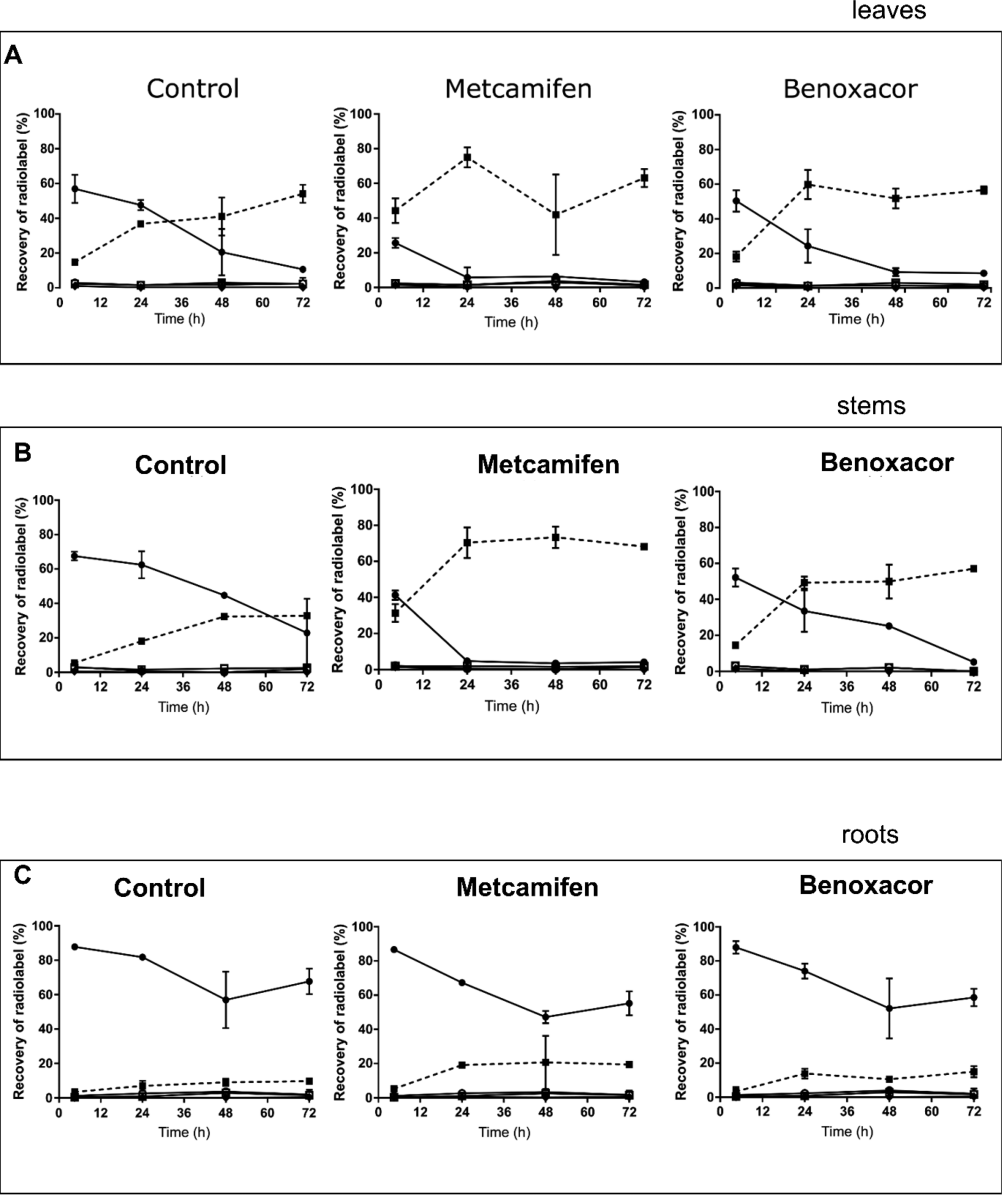
Metabolism and disposition of [^14^C]-mesotrione in maize. Each point represents the mean ± SD (*n* = 3) of the abundance of the parent herbicide (●) and metabolites 4-hydroxymesotrione (■), 5-hydroxymesotrione (⧫), and 4-methylsulfonyl-2-nitrobenzoic acid (MNBA) (○) in leaves (A), stems (B), and roots (C) pretreated with the solvent carrier alone, metcamifen, or benoxacor. Levels of the metabolite 2-amino-4-methylsulfonyl-benzoic acid (AMBA) and the uncharacterized polar metabolites were negligible in all tissues examined over the period of this study.

The fate of the herbicide was studied in leaves (Figure 3A), stems (Figure 3B), and roots (Figure 3C), with the formation of the respective radioactive metabolites quantified by radio-TLC. Over the course of this short metabolism study, near quantitative (100%) recovery of the applied radioactivity was recorded as partitioned between the plant tissue and liquid medium. [^14^C]-Mesotrione was rapidly taken up into the roots and translocated to the stems and leaves, with the presence of the safeners accelerating the disappearance of the parent herbicide in the treated plants in the order of metcamifen > benoxacor > control, with the concomitant appearance of radioactive metabolites, notably 4-hydroxymesotrione (Figure 3A–C). Smaller amounts of MNBA as well as 5-hydroxymesotrione were also determined in all three tissues, with the accumulation of these alternative CYP products enhanced by treatment with metcamifen but not with benoxacor. These studies demonstrated that in maize, safeners selectively enhance specific CYP-mediated biotransformations, notably the 4-hydroxylation of mesotrione, in favor of oxidative cleavage, or 5-hydroxylation. The experiment also demonstrated that with mesotrione as the herbicide partner, metcamifen was a more effective inducer of CYP-mediated detoxification than benoxacor.

### Identification of Safener-Inducible Genes with Potential Roles in Herbicide Selectivity

**Figure 4:**
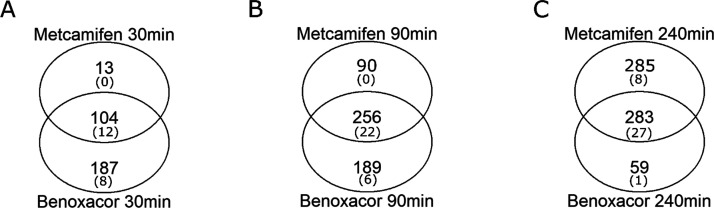
Venn diagram of global transcript enhancement in maize suspension cultures treated with metcamifen or benoxacor for (A) 30 min, (B) 90 min, and (C) 240 min as compared with cells treated with DMSO. Upregulated detoxification genes including CYPs, glutathione transferases (GSTs), and ABC transporters (ABCs) within each group are collectively shown in parentheses.

To identify which genes are linked to herbicide safening in maize, an RNaseq experiment was carried out using BMS cell suspension cultures treated with metcamifen or benoxacor as compared to control samples treated with solvent carrier only. BMS cultures were used instead of whole plants as a source of maize cells responding synchronously to the safener treatment, without the need to account for bioavailability issues arising from uptake and translocation. Following assembly, a total of 34 958 gene sequences were identified. The treatments were considered to have significantly altered gene expression if the respective unigenes showed alterations in abundance of 2-fold or greater (FDR < 0.05). There was considerable overlap of genes significantly upregulated by metcamifen and benoxacor, with just over a third of transcripts induced by both safeners at 30 and 90 min, rising to 45% at 240 min (Figure 4). Benoxacor induced more transcripts at the earlier time points, but at 240 min, metcamifen was the most effective enhancer (Figure 4). The ontology analysis of these contigs yielded 11 060 GO terms relating to biological processes, molecular functions, and cellular components. GO term genes showing changes in abundance (*p*-value ≤ 0.05) on safener treatment were further analyzed (Table S3). The GO term genes related to NAD+ ADP-ribosyltransferase activity anchored components of the plasma membrane, and oxidation–reduction processes were enhanced by benoxacor treatment. On the other hand, the GO gene terms linked to the cellular response to chemical stimulus, chloroplast, and transferase activity were induced by metcamifen. Interestingly, both safeners repressed GO terms linked to isomerase activity.

**Figure 5:**
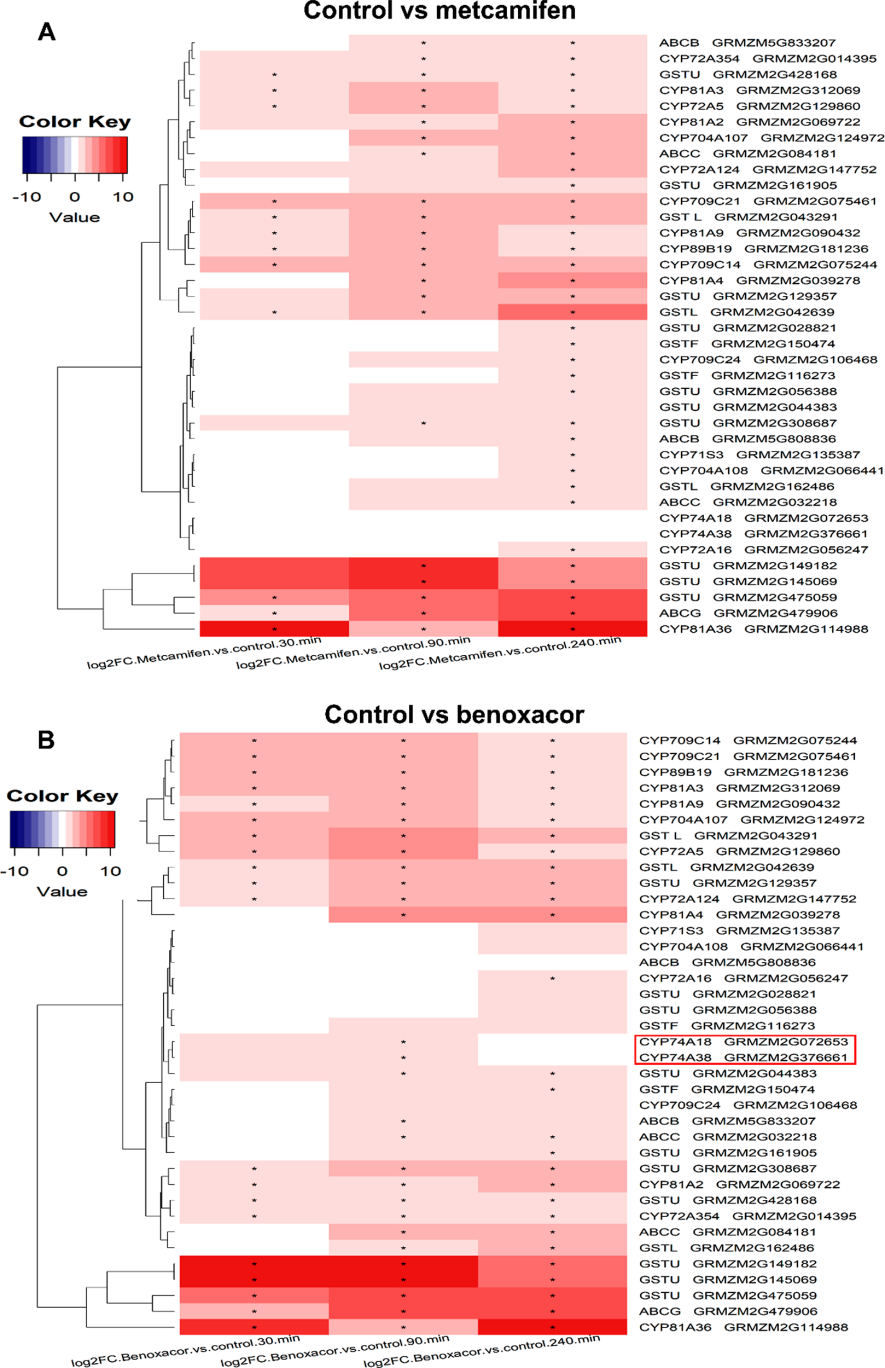
Heatmap representing the log_2_ fold changes of xenome genes from CYP, GST, and ABC families in BMS cells treated with (A) metcamifen or (B) benoxacor compared with DMSO control treatments. The red coloration indicates the upregulation levels of gene induced by safeners, * designates an FDR ≤ 0.05. Transcripts were identified based on the respective standard nomenclature with the respective unigene sequences corresponding to those in the maize genome databases.

Overall, 6% of safener-induced transcripts were associated with herbicide detoxification (xenomes), including representatives of the CYP, GST, and ABC transporter superfamilies. Primary analysis showed that the majority of induced xenome genes were enhanced by both safeners, with benoxacor being more active at the earlier time points (Figure 4A). The expression of each family of xenome genes in response to the two safeners was then subject to a heatmap analysis at 0, 90, and 120 min time points (Figure 5). This analysis identified the safener-mediated induction of 18 CYPs, 3 lambda GSTs (GSTLs)s, 2 phi GSTs (GSTFs), 10 tau GST (GSTUs), and 5 ABC proteins, with metcamifen and benoxacor promoting similar patterns of induction across the gene families. The only exception was with two CYPs in family 74, which were exclusively upregulated by benoxacor at 90 min (Figure 5B).

Further analysis of the 18 safener-induced CYPs (Table S4) showed that members of clan 71 and clan 72 were the most responsive. On applying a search for genes consistently induced from 30 to 240 min by both benoxacor and metcamifen, CYP81A3, CYP81A9, CYP81A36, and CYP89B19 from clan 71 and CYP72A5, CYP72A124, CYP709C14, and CYP70921 from clan 72 were identified as being most safener-responsive in maize. Most CYPs were induced less than 20-fold, except CYP81A36, which was hypersensitive to safener treatment. The 18 upregulated CYPs (Table S4) were then aligned to a phylogenetic tree constructed from 270 maize CYP sequences previously identified and classified using a uniform nomenclature.^12^ These safener-responsive CYPs were then prioritised for functional characterization of their herbicide detoxification activities using recombinant yeast systems.

### *In Vivo* Screening of Recombinant Maize CYPs for Activities toward Herbicides

The ORFs of CYPs induced by safeners in maize cultures were codon-optimized for expression in yeast and cloned into pYES3/CT vectors for the functional characterization of the respective recombinant enzymes. Yeast strain WAT11, which coexpresses an *Arabidopsis*-derived CYP reductase, was used as the host cell system to increase the likelihood of successful functional expression of plant CYP activity.^23^ As maize CYPs act on a diverse range of herbicide chemistries (Figure S1), it was necessary to identify a small number of candidates that could be used for primary recombinant enzyme screening and assay development. The two herbicides selected were nicosulfuron and bentazon, which represented diverse chemistries and undergo well-characterized and -defined CYP-mediated biotransformations. Importantly, their CYP-based detoxification in maize is also genetically linked to the selectivity of a wider range of herbicides including mesotrione,^30^ which was the subject of our earlier metabolism studies (Figure 3). Nicosulfuron and bentazon were individually fed to the induced yeast cultures at a final concentration of 25 μM. After incubating for 24 h, the supernatant was analyzed by LC–MS, and the mass spectra of resolved compounds were interrogated for oxidative reaction products (Table S1). Out of the 20 CYP-transformants tested, the presence of hydroxylated bentazon (parent [M – H]^−^ 239, metabolite [M – H]^−^ 255) was determined in eight cultures, with the highest level of product accumulation seen with clan 71 enzymes, notably CYP81A1, CYP81A2, CYP81A4, CYP81A9, and CYP81A16 (Table S5). In contrast, the oxidative biotransformation of nicosulfuron (parent [M–H]^+^ 411, metabolite [M–H]^+^ 427) was not detected in any of the cultures. Further investigation with the respective yeast microsomes showed this lack of nicosulfuron metabolism was due to an apparent lack of uptake of the herbicide into the yeast cells, probably as a consequence of its physicochemical characteristics. To avoid further false negatives, definitive activity testing of recombinant CYPs (rCYPs) in all other herbicides tested was performed *in vitro*, using the respective yeast microsomes.

### Development of Recombinant CYP Metabolism Assays

A series of herbicides used in maize and representing a diversity of chemistries metabolized by CYPs were assembled for testing (Figure S1), with a literature search performed to define a library of probable, or predicted, oxidation products that could be detected and identified by ESI MS (Table S1). To optimize rCYP activities, bentazon and nicosulfuron were selected to establish robust and quantitative assay conditions for the recombinant enzymes following their expression in yeast microsomes. CYP81A9 was selected as the test enzyme, as earlier classical genetic studies had identified the respective gene, designated *nsf1* or *ben1*, as controlling sensitivity to seven classes of herbicide that are all detoxified by CYPs.^31−33^ CYP81A9 was expressed in microsomes prepared from WAT11 yeast cells following high-density culturing.^24^ In an attempt to quantify enzyme expression, maize rCYPs were treated with carbon monoxide to generate the respective heme cofactor complex associated with a quantitative Soret spectral shift at 450 nm.^34^ While a Soret peak could be readily detected at 450 nm with an expressed human rCYP51 (data not shown), the complex could not be detected in any of the maize rCYP microsome preparations, suggesting relatively low levels of expression of the recombinant proteins.^34^ As an alternative approach to quantify the rCYP protein content in yeast microsomes, an MS-based targeted proteomics approach was taken. A unique isotopically labeled peptide standard derived from CYP81A9 was synthesized and used to quantify the respective tryptic fragment released from the digestion of the respective microsome preparation. While this methodology could not predict the amount of functionally active CYP present, it did allow for the more accurate comparison of specific activities toward the herbicide substrates tested between different enzymes. Using this method, CYP81A9 was found to be expressed as 40 pmol of rCYP mg^–1^ of total microsomal protein (Table S6).

Yeast microsomes expressing rCYP81A9 were then incubated with bentazon or nicosulfuron in the presence and absence of the cofactor NADPH, with the formation of oxidative metabolites determined by LC–MS (Table S1). Hydroxylation products from both herbicides were tentatively identified and further characterized by a combination of accurate mass determination (<5 ppm) and MS–MS fragmentation analysis. By reference to the literature, these analyses confirmed that nicosulfuron was hydroxylated on the pyrimidinyl ring, most likely at the 5-position,^35^ while authentic reference standards confirmed the bentazon product to be 6-hydroxybentazon. Using the two herbicides, assays were then optimized with respect to the dependence of product formation on incubation time, protein concentration, and substrate concentration (Figure S3). The optimal conditions determined were a 20 min incubation period, with 200 μM herbicide using microsome extracts prepared as 2.5 mg of crude microsomal protein mL^–1^ per assay.

### Activities of Safener-Inducible Maize CYPs toward Herbicides

Members of the clans 71, 72, 74, and 86 CYPs, identified as being safener-inducible (Table S4), were mapped to the phylogenetic tree of known maize CYPs (Figure S4).^12^ The safener-inducible CYPs were then assayed using the whole cell metabolism screens with bentazon and then additionally as the respective microsome preparations with nicosulfuron and the triketone herbicides mesotrione and tembotrione. Using bentazon as the substrate, with the exception of CYP72A124, oxidative activity toward the herbicides was almost exclusively restricted to the clan 71 CYPs (Table S5). Attention was therefore focused on the eight CYP81A family members associated with safening in clan 71. All CYP81As (except CYP81A1, which could not be quantified for technical reasons), expressed in yeast, with levels varying from 0.5 to 67 pmol mg^–1^ of yeast microsomal protein (Table S6). The levels of expression of this group of recombinant proteins were found to be consistent between batches, though much lower than the yields of 100–400 pmol of rCYP mg^–1^ of microsomal protein reported for mammalian rCYPs.^24^ For each herbicide, rCYP activity caused the hydroxylation of the parent compound, with oxidation occurring on the pyrimidine ring of nicosulfuron, at the 6- or 8-positions of the carbocyclic ring of bentazon and at the 4-position of the cyclohexanedione rings of mesotrione and tembotrione, respectively. Four of the expressed enzymes (CYP81A1, CYP81A3, CYP81A17, and CYP81A36) were inactive toward the four herbicides, while CYP81A4 showed limited activity toward bentazon only. The remaining three enzymes showed a spectrum of related activities apparently linked to their sequence relatedness (Figure S4), with CYP81A9 and CYP81A16 sharing 97% amino acid identity (Figure S5), showing similarly high activities to all four herbicides. In contrast, CYP81A2 with 77% identity to CYP81A9 had a distinct activity profile toward the four herbicides tested, being the only CYP able to hydroxylate bentazon at the 8-position. On performing basic kinetic analyses, all three CYP81As showed low binding specificity toward the herbicides with saturable kinetics only shown with bentazon at concentrations exceeding 200 μM substrate (Figure S6).

CYP81A2 and CYP81A9 showed 77% identity, but distinct activity profiles were exploited using a wider range of herbicides used in maize (Table 1). Both enzymes were similarly active toward bentazon, the phenylurea chlortoluron and the ALS inhibitors chlorimuron ethyl, triasulfuron, and flumetsulam. Unlike CYP81A9, CYP81A2 showed no activity toward triketones, imidazolinone, and most of the pyrimidnylsulfonylurea herbicides. Considering the HPPD inhibiting herbicide triketones as structurally related substrates, while CYP81A9 hydroxylated all the benzoylcyclohexanedione compounds, with particularly high levels of activity observed toward tembotrione, CSCC152531, and CSAA464664, the substitution of the cyclohexanedione with a pyrazole ring (topramezone) resulted in a loss in activity. With the ALS inhibitors, CYP81A9 catalyzed the hydroxylation of the pyrimidinyl rings of nicosulfuron, rimsulfuron, and foramsulfuron (Table 1). Replacement of one of the methoxy group substituents of the pyrimidinyl ring with Cl (chlorimuron ethyl) resulted in a 10-fold loss in activity, which was further reduced by substitution with OF_2_ (primisulfuron-methyl), which led to hydroxylation on the phenyl ring. Similarly, substitution of the pyrimidine with a triazine resulted in hydroxylation on the phenyl ring of triasulfuron by both CYP81A9 and CYP81A2, with flumetsulam hydroxylated on the difluorophenyl ring by both enzymes. Of the three imidazolinone herbicides tested, activity was only observed with CYP81A9 toward imazethapyr (Table 1).

**Table 1:** Comparison of CYP81A9 and CYP81A2 Specific Activities^a^ toward the Range of Maize Herbicides Shown in Figure S1^b^

	Specific activities (pkat mg^–1^ recombinant CYP)	
Substrate	CYP81A9	CYP81A2
***Triketones (HPPD inhibitors)***		
Mesotrione	**665** ± 38	0
Sulcotrione	**2028** ± 358	0
Tembotrione	**6791** ± 320	0
CSCC152531	**9648** ± 1501	0
CSAA464664	**12 869** ± 4692	0
***Benzoylpyrazole (HPPD inhibitor)***		
Topramezone	0	0
***Pyrimidinylsulfonylureas* (*ALS-inhibitors*)**		
Nicosulfuron	**909** ± 167	0
Rimsulfuron	**691** ± 79	0
Foramsulfuron	**1199** ± 17	0
Chlorimuron-ethyl	**74** ± 18	**50** ± 3
Primisulfuron-methyl	**37** ± 4	0
***Triazinylsulfonylureas(ALS inhibitors)***		
Triasulfuron	**109** ± 15	**62** ± 11
***Sulfonanilide* (*ALS inhibitors*)**		
Flumetsulam	**143** ± 41	**33** ± 9
***Imidazolinone (ALS inhibitor)***		
Imazethapyr	**153** ± 26	0
Imazamox	0	0
Imazaquin	0	0
***Phenylureas (PSII inhibitors)***		
Chlorotoluron	**30** ± 5	**46** ± 3
Isoproturon	0	0
Linuron	0	0
***Chlorotriazine (PSII-inhibitor)***		
Atrazine	0	0
***Cyclohexene oxime (ACCase-inhibitor)***		
Sethoxydim	0	0
***Thiadiazine (Growth regulator)***		
Bentazon	**2221** ± 406*	**1579** ± 278*
	0^#^	**251** ± 41^#^

a Mean ± SD, *n* = 3.

b In the case of bentazon, two activities were detected, notably hydroxylation at the 6- and the 8-positions as denoted by * and ^#^, respectively.

### Exploring the Herbicide Detoxifying Activity of Family CYP81A through Protein Engineering

**Figure 6:**
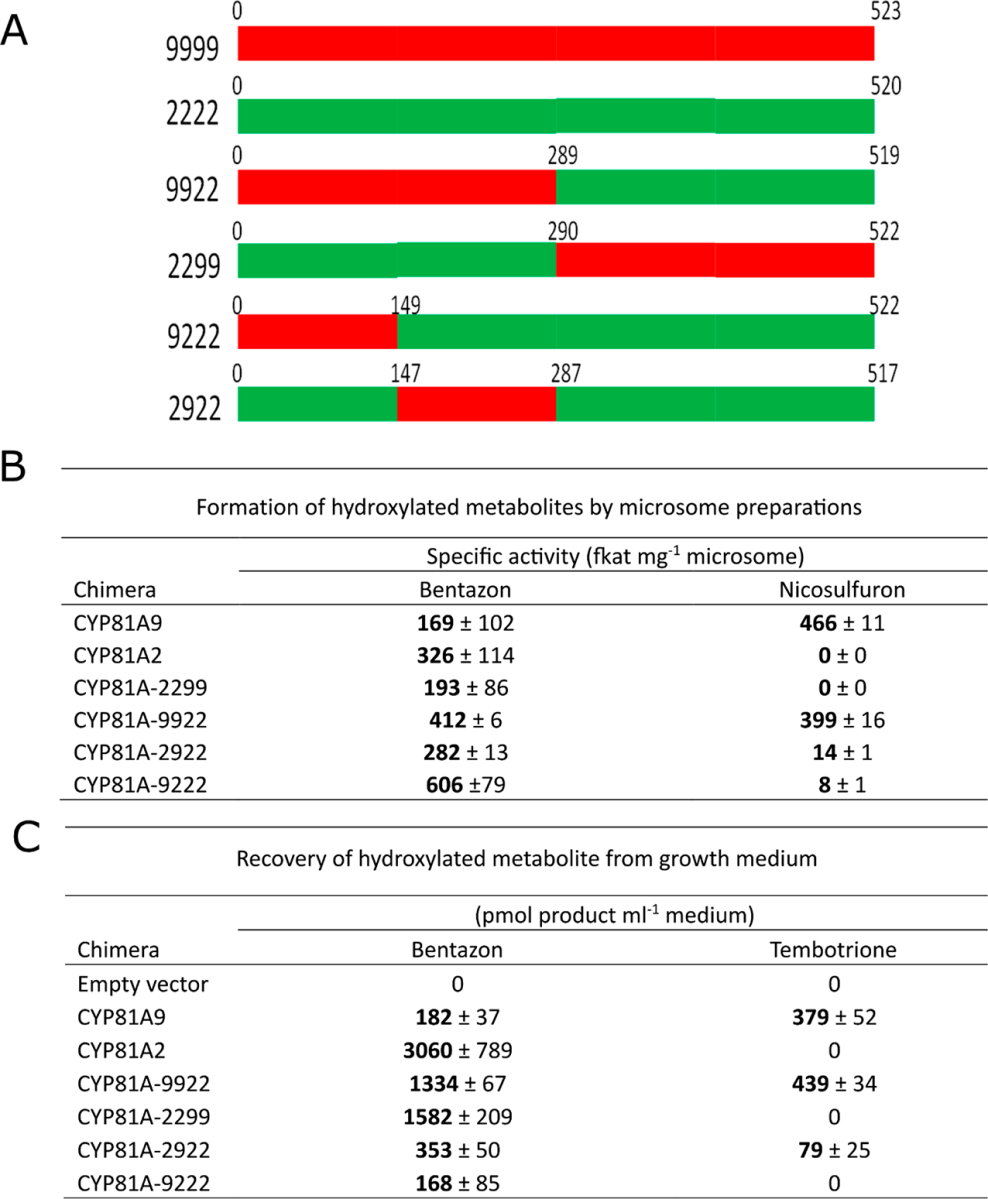
Engineering of maize CYP81A activities by gene splicing of family members. (A) Recombinant chimeras were constructed by splicing together blocks of the sequences of CYP81A9 (shown in red) and CYP81A2 (green). The chimeras were then assayed for enzyme activity toward bentazon (6- and 8-hydroxylation), nicosulfuron (pyrimidinyl hydroxylation), and tembotrione (4-hydroxylation) using either (B) microsome preparations or (C) yeast culture feeding assays. Mean ± SD of enzymatic activity of three biological replicates (*n* = 3) were reported.

Within the CYP81A family, the observation that closely related enzymes showed differing activities toward a panel of herbicides provided a basis for structure–function relationships to be carried out. Thus, while CYP81A9 acted on a diverse range of herbicide chemistries and showed high activities toward the triketone series, the closely related CYP81A2 was more constrained, being unable to hydroxylate the HPPD inhibitors or sulfonylureas. However, uniquely within this subfamily, CYP81A2 was able to catalyze the ring hydroxylation of bentazon at both the 6- and 8-positions (Table 1). To explore the basis of the respective activities of CYP81A9/A2, the substrates defining this selectivity were selected as bentazon, nicosulfuron, and tembotrione. A SWISS-MODEL program from ExPasy was then used to build structural models of CYP81A9 and CYP81A2 using Steroid 17-α-hydroxylase/17,20 lyase (4nkv.3.A, human steroidogenic cytochrome P450 17A1 mutant A105L with inhibitor abiraterone) as a template (Figure S7A). Using this model, chimeric enzymes composed of blocks of sequence from CYP81A2 and CYP81A9 were then assembled, with splicing points chosen that were predicted to be contained within flexible loops on the outside of the protein, rather than within defined core secondary structures (Figure S7B), such as helices or putative substrate recognition sites (SRSs).^36^ SRSs were potentially of particular interest, being short blocks of sequence motifs linked to the selectivity of CYP binding to their acceptor substrates.^36^ The modeled chimeras (Figure S7B) were then named based on their assembly from quarter length blocks of sequence (Figure 6).

In the first instance, chimeras were generated from splicing the two halves of the respective coding sequences together to generate reciprocal enzymes composed of the N-terminal of CYP81A2 (termed CYP81A-2299) or CYP81A9 (CYP81A-9922), respectively (Figures 6A and S7B). The associated CYP activity was then assessed by a combination of testing microsome preparations (Figure 6B; bentazon and nicosulfuron) or, in an attempt to increase assay sensitivity, by feeding to the respective recombinant yeast cultures (Figure 6C; bentazon and tembotrione). Using these two assay systems, activity toward bentazon was observed with both chimeras, but only CYP81A-9922 was able to hydroxylate nicosulfuron and tembotrione, indicating the N-terminal half of CYP81A9 conferred activity toward these herbicides (Figure 6B,C). Further splicing of the N-terminal domain of CYP81A9 generated a further two chimeras CYP81-9222 and CYP81-2922, both of which showed reduced activity toward bentazon and a barely detectable ability to hydoxylate nicosulfuron as compared with CYP81A-9922. Of the second-generation chimeras, CYP81A-2922 was active toward tembotrione, albeit less so than the 9922 chimeras, while the reciprocal CYP81A-9222 was not, revealing that much of the critical activity toward tembotrione was conferred within the second quartile of the CYP sequence, corresponding to amino acids 147–290. This quarter is predicted to contain helices F and G, which in turn contain putative SRS2 and SRS3, respectively (Figure S7A,B). Previous research on CYPs with solved structures suggests that the F-G helical bundle participates in substrate binding and controls access to the active site,^37^ with mutations in this domain resulting in a dramatic reduction in oxidative activity toward substrates.^38^ Comparison of the F-G helices region between CYP81A9 and CYP81A2 revealed seven amino acid differences (Figure S7C). Two of these amino acids at T207 and G269 in CYP81A9 could be discounted as being related to substrate selectivity, as CYP81A16, which is effectively the twin of CYP81A9, contains the same amino acids at these positions as CYP81A2 (Figure S7C). To assess the importance of the amino acids that did differ between CYP81A9 and CYP81A2, two new constructs based on the sequence of CYP81A2 were designed. The first, covering three amino acid differences, replaced the SRS2 sequence for CYP81A2 with that of CYP81A9, and the second construct contained the mutation TA255LD in helix G near to SRS3 of CYP81A2 (Figure S7C). In both cases, the parent CYP81A2 sequence was engineered to express the two respective sets of mutated residues to effectively determine whether such modifications could confer selectivity toward herbicides other than bentazon. Following their expression in yeast, the cultures were fed with either bentazon or tembotrione. While both mutants were active toward bentazon, no oxidized metabolites of tembotrione could be detected (data not shown), suggesting that the individual putative SRS2 and SRS3 domains of CYP81A9 were not sufficient in themselves to selectively confer activity to triketone herbicides. Instead, it is possible that these domains have a more general functional activity in influencing the accessibility of hydrophobic herbicide substrates to the CYP active site.

### CYP Protein Expression in Maize Suspension Culture and *in Planta*

To better define the relative importance of different CYPs in herbicide safening, microsomes were prepared from BMS suspension cultured cells treated with or without 5 μM metcamifen and assayed in the presence of NADPH with the herbicides nicosulfuron, mesotrione, and bentazon. Oxidative reaction products were detected for all three substrates, with safener treatment enhancing the respective biotransformation reactions in each case between 2- and 3-fold (Table S7). In the case of bentazon, 6-hydroxylation > 8-hydroxylation reactions were determined, while with mesotrione, only 4-hydroxylation was observed, consistent with the dominant route of metabolism determined with this herbicide in safener-treated maize (Figure 2). These microsomes were then subjected to tryptic digest and proteomic analysis for membrane-associated polypeptides and for their relative change in abundance following metcamifen treatment (Table S8). Some 31 microsomal proteins were significantly perturbed by safener treatment, with 17 upregulated some 1.5-fold and 14 downregulated around 2-fold (Table S8). The identities of the proteins revealed a mixture of functions linked to both primary and secondary metabolism. With respect to xenobiotic metabolism and detoxification, safener-enhanced CYPs, the Lambda GST In2-1, and the three ABC transporter proteins were of particular interest. Further interrogation of the tryptic fragments revealed the presence of 11 distinct CYPs, of which 6 had been identified in the transcriptome experiment. Of all the upregulated CYPs, CYP81A9 was the most strongly induced (2.3-fold), followed by CYP81A3 (1.6-fold). The change in abundance of the other CYPs, which were mainly from clans 71 and 72, were not judged as being significant and included CYP81A16, which is identical to CYP81A9, as well as CYP72A16, CYP89B19, and CYP704A108, all of which were upregulated by metcamifen at the transcript level (Table 2).

**Table 2:** CYP Protein Abundance Identified by Proteomics Using Microsomes Prepared from BMS Cultures Treated with or without 5 μM Metcamifen^a^

CYP name/description	Protein ID	Fold change	Unique peptides
CYP72A16	Q8LL74	1.258	6
CYP81A9	B6SSF2	2.261^b^	10
CYP81A3	B4G1A3	1.639^b^	15
CYP714B10	A0A1D6F4G3	1.065	14
CYP721B4-like	B4FTC5	0.904	2
Putative cytochrome P450 superfamily protein	A0A1D6G961	0.997	2
CYP89B19	A0A1D6KQU8	0.938	3
CYP72A123	B6SXS5	1.072	18
CYP71K15	B6TWF0	1.020	8
CYP704A108	C0PCX4	0.948	6

a The fold change represented protein abundance in the metcamifen treatment.

b Statistically significant change.

Having identified safener-inducible CYP81As in BMS cultures, it was then of interest to determine whether these genes were also enhanced by similar exposure in whole seedlings. Hydroponically grown maize seedlings were exposed to a 1 h pulse treatment with 5 μM metcamifen via the growth medium and then harvested 2 h later. The treatment regime was chosen to capture the rapid xenobiotic response (RXR) invoked in plants that show herbicide safening.^3,8^ Real-time qPCR was performed for each member of the CYP81A subfamily, which had been characterized with respect to enzyme activity, with double strand (ds) DNA fragments of each CYP being used to create the standard curve for absolute quantification of the transcript abundance of each CYP (Table 3). Compared to other CYP81As, only very low levels of CYP81A1, CYP81A3, and CYP81A17 transcripts were detected as being constitutively expressed in both stem and leaf. CYP81A1 and CYP81A17 were also unique in showing no enhancement in transcript abundance following metcamifen treatment. For the other CYP81As, constitutive levels of expression were generally higher in the leaves than the stems, though in terms of fold induction, the stem tissue was more responsive to safener treatment. The exception was CYP81A36, which was present at much higher levels in stem tissue whether safener-treated or not, mirroring its abundance in the BMS cultures. By comparison with the safening observed in the BMS cultures following a 2 h exposure, the induction of CYP81A3 and CYP81A9 was greater in the stems than in the cultures, while CYP81A4 and CYP81A2 were similarly responsive in the two test systems.

**Table 3:** Absolute Quantification of CYP81A Transcripts^a^ in Hydroponically Grown Maize Seedlings (cv. Garland) Maize Treated with ±5 μM Metcamifen for 1 h via the Growth Medium and Then Harvested after a Further 2 h^b^

	Transcript abundance (Transcript number ng^–1^ mRNA)			
	Stem		Leaf	
Gene	Control	Metcamifen	Control	Metcamifen
CYP81A1	**98** ± 58	**75** ± 45	**42** ± 31	**52** ± 17
CYP81A2	**208** ± 170	**1453** ± 148	**392** ± 101	**1889** ± 482
CYP81A3	**54** ± 25	**536** ± 102	**6** ± 0	**20** ± 5
CYP81A4	**637** ± 109	**3449** ± 784	**2312** ± 883	**5673** ± 690
CYP81A9	**392** ± 131	**5026** ± 868	**1126** ± 370	**4298** ± 872
CYP81A16	**211** ± 102	**3835** ± 597	**521** ± 220	**3217** ± 801
CYP81A17	**24** ± 7	**25** ± 5	**41** ± 21	**4** ± 20
CYP81A36	**698** ± 100	**8093** ± 1620	**174** ± 36	**1853** ± 447

a Mean ± SD, *n* = 3.

b Absolute quantification of transcript abundance was determined by real-time qPCR using a standard curve of amount double stranded DNA fragments of each CYP.

## Discussion

Herbicide safeners are an increasingly important group of agrochemicals that allow existing postemergence herbicides to be used in new applications for selective weed control in crops that would otherwise suffer unacceptable chemical damage.^2^ In cereals such as maize, safener activity is integrally linked to enhanced herbicide metabolism in the crop and associated with the enhanced transcription and expression of key detoxifying enzymes.^2,7^ While in previous studies in rice, we had determined that different safener chemistries each invokes unique changes in gene expression;^8^ in this study, benoxacor and metcamifen promoted very similar changes in the respective transcriptomes in maize. Genes linked to herbicide detoxification were strongly represented (6%) among the respective safener-induced transcripts and included GSTs and ABC transporters in addition to CYPs. The enhancement of the ABC proteins was particularly interesting, given their known role in xenobiotic detoxification in animals but largely unstudied role in herbicide metabolism and safening.^4^ With respect to CYPs, safening in maize was associated with the activation of 18 gene family members, drawn from 4 distinct clans, notably clans 71 and 72. Functional analysis of these inducible CYPs using a recombinant yeast expression system revealed that just three members of CYP81As within clan 71 were predominantly responsible for the detoxification of a structurally diverse range of maize herbicides. Two of these enzymes, CYP81A9 and CYP81A16, were 97% identical and showed a similar spectrum of activity. In contrast, CYP81A2 showing 77% identity to CYP81A9 showed a more restricted range of activities, notably toward triketone and sulfonylurea chemistries, but was unique among the CYPs identified in hydroxylating bentazon in both the 8- and the 6-positions. Attempts to probe the structural specificity of the activities of CYP81A9 and CYP81A2 through the construction of enzyme chimeras showed that the ability to hydroxylate the triketone tembotrione resided in the N-terminal portion of CYP81A9, notably in the second quartile. However, more detailed dissection of the second quartile of CYP81A9 failed to identify critical residues responsible for this switch in specificity and in any role for the putative substrate recognition site residing in the respective domain.^37^

In terms of their contribution to the safener-inducible metabolism and detoxification of herbicides, the combination of quantitative transcriptomics and proteomics and functional enzyme characterization clearly identified CYP81A9 and its homologue CYP81A16 as being of critical importance. The role of CYP81A9 in herbicide detoxification in maize had previously been identified through classical genetic studies. Thus, sensitivity to seven classes of herbicide chemistry in sweet corn inbred hybrids was traced to a recessive gene designated as either *nsf1* or *ben.1.*(31−33) The herbicide sensitivity trait in sweet corn linked to *nsfI* was subsequently mapped to CYP81A9.^33^ In contrast, the potential importance of CP81A16 has not been reported. In the current study, while CYP81A16 was identified in the NCBI database, the corresponding unigene was not identified in the RNaseq data from BMS cultures. Gene-specific PCR studies with genomic DNA subsequently showed that that an amplicon of 179 bp corresponding to CYP81A9 could be identified in BMS cultures. However, in seedlings of the sweet corn cultivar Sundance and the field maize cultivars Garland and Maxxis Duo, the 102 bp amplicon of CYP81A16 was only present in the field maize cultivars (Figure S8). Quantitative PCR suggests that transcripts encoding CYP81A9 and CYP81A16 were similarly abundant in field maize cultivars (Garland), being equally responsive to metcamifen treatment (Table 3). This suggests that while CYP81A9 is predominantly responsible for herbicide metabolism in sweet corn hybrids, this activity is shared with CYP81A16 in field maize. Such an observation is consistent with the duplication of the parent gene occurring after the domestication of sweet corn from the lineage leading to field maize varieties.

**Figure 7:**
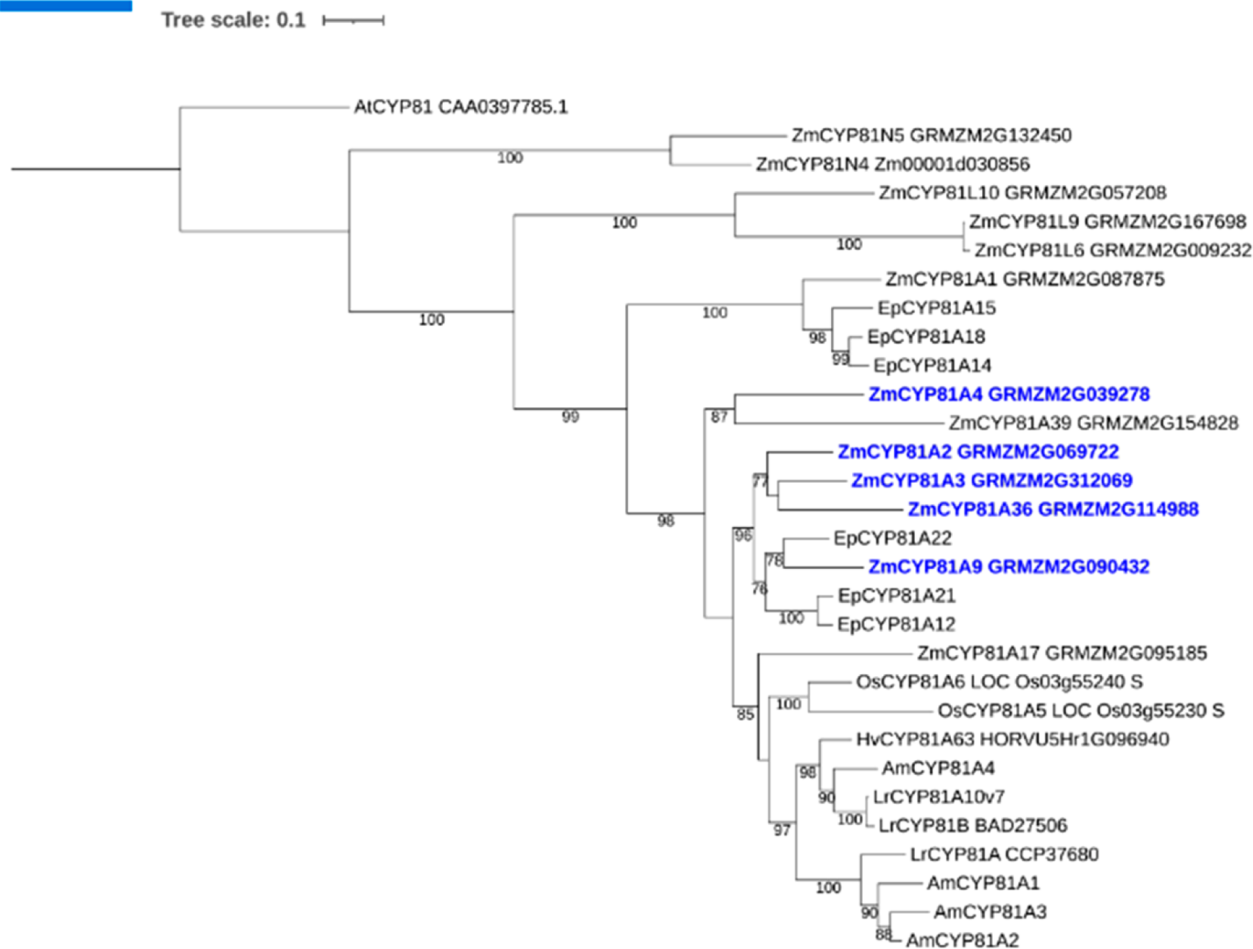
Phylogenetic analysis of CYP81 proteins from crop plants and wild grasses. Amino acid sequences of maize (Zm, *Zea mays*), rice (Os, *Oryza sativa*), wheat (Ta, *Triticum aestivum*), black grass (Am, *Alopecurus myosuroides*), late watergrass (Ep, *Echinochloa phyllopogon*), annual ryegrass (Lr, *Lolium rigidum*), and barley (*Hv*, *Hordeum vulgare*) were used for maximum likelihood alignment for phytogenic analysis. The number on the branch represents the bootstrap support values above 75%. The scale bar indicates the inferred number of substitutions per site. Sequences in blue correspond to transcripts from maize upregulated by the safeners metcamifen and benoxacor. The tree was rooted using the sequence of *At*CYP81_ CAA0397785.

A phylogenetic analysis of the safener-inducible maize CYP81s as aligned to related enzymes in other cereals and wild grasses was then constructed (Figure 7). This analysis showed that CYP81A9 clustered closely with CYP81A12 and CYP81A21 from late water-grass (*Enchinchloa phyllopogon*), which are also active toward multiple herbicides.^39^ This small group of related CYP81As is therefore of particular interest, as they have evolved from a common progenitor primed to detoxify herbicides long before the exposure of grasses to synthetic chemistries. In contrast, in another evolutionary branch of the superfamily, CYP81A17, which showed no activity toward herbicides tested in this study, clustered with *Lr*CYP81A10v7 from annual ryegrass (*Lolium rigidum*), an enzyme linked to the hydroxylation of diclofop methyl and mesotrione in herbicide-resistant populations.^40^ Other notable CYP81As from other species that align to the maize enzymes include CYP81A14, CYP81A15, and CYP81A18, which can be induced by the application of bispyribac sodium in late water-grass,^41^ and CYP81A10v7, which is upregulated in resistant annual ryegrass populations.^40^ The dendogram also identified the rice CYP81A6 as being related to maize CYP81A9 (73% identity). Rice CYP81A6 was originally mapped to a gene responsible for conferring tolerance to bentazon and sulfonylurea herbicides.^42,43^ As is the case with CYP81A9 in maize, the CYP81A6 gene was highly induced (30-fold at 90 min) in rice cell cultures exposed to metcamifen.^8^

Collectively, these studies further point to the central importance of a small number of clan 71 CYPs from the CYP81 family as being instrumental in herbicide metabolism in crops and weeds. Intriguingly, the CYP81s of maize have also recently been shown to display a range of overlapping activities involved in the biosynthesis of antimicrobial natural products known as zealexins.^44^ Based on the large size of the CYP superfamily in higher plants, this specialization in the metabolism of xenobiotics in such a small number of CYP81s is perhaps surprisingly similar to the situation in mammals, where the ability to metabolize drugs in humans is limited to CYPs from families 1, 2, and 3.^45^ We postulate there are two major drivers for the development of such detoxification traits in higher plants. First is in protection against allelochemicals produced by competing plants or fungal or bacterial phytotoxins produced by necrotrophic pathogens. Second is in protecting the plant host from autotoxicity caused by the production of its own reactive chemical defenses such as phytoalexins and oxidized fatty acid derivatives. Since these toxins are likely to be highly diverse in their chemistries, the observed low specificity of the maize CYPs to the synthetic compounds used in the current study points to a promiscuity in specificity as has been determined in human-drug-metabolizing CYPs.^46^ The fact that herbicide detoxification in cereals appears to reside in a small number of closely related CYP81As is surprising based on the clear differences in the biotransformation capacity of the different crop species toward different selective herbicides. Taken together with the known key role for CYPs in the evolution of non-targeted site herbicide resistance in wild grasses,^14^ this does suggest that that the CYP81A enzyme scaffold has the potential for considerable plasticity in its ability to evolve new detoxifying activities toward different herbicide chemistries.

Finally, while the current study sheds light on the role of clan 71 CYPs in safener-inducible herbicide metabolism in maize, the roles of the other CYPs enhanced by exposure to these chemicals is unclear. Certainly, in other cereals the metabolism of specific herbicides has been linked to clan 72 enzymes. For example, in rice, CYP72A18 has been shown to hydroxylate pelargonic acid,^47^ while map-based cloning identified CYP72A31 as responsible for the tolerance to the ALS inhibitor bispyribac sodium in *Oryza indica.*(48) However, it is clear that the majority of the safener induced CYPs do not have activity toward herbicides, begging the question as to their roles in endogenous metabolism and functional links to the safener response. Besides detoxification, many CYPs are known for their functions in plant secondary metabolite biosynthesis. For example, CYP members of clans 71 and 72 are involved in the biosynthesis of flavonoids and terpenoids.^49,50^ Intriguingly, safener treatment of wheat is known to be associated with the accumulation of a range of flavone derivatives.^51^ Similarly, while two proteins from clan 74 (CYP74A38) and clan 86 (CYP 704A108) were upregulated by safeners, neither CYP showed activity toward any of the herbicides tested. However, it is perhaps significant that enzymes related to these family CYP704 and family CYP74 proteins have roles in the biotransformation of fatty acids linked to plant vigor and stress-related signaling, respectively.^52,53^ It is therefore possible that while not involved directly in herbicide detoxification, several safener-induced CYPs are more involved in endogenous stress response signaling pathways that lead to phytoprotection by alternative routes.^54^

## Materials and Methods

### Plant Studies

Both benoxacor and metcamifen are typically coapplied by spray application with their herbicide partners. In the experiments described here, safeners and herbicides were coapplied in aqueous treatment media in lieu of spray application. Based on delivering functional safening activity, this route of application has been shown to give similar protective activities to those delivered through formulated spray applications or seed treatment in both maize and rice.^7,8^ Black Mexican Sweet Corn (BMS) suspension cell cultures were grown and maintained as described previously.^8^ At 5 days after subculturing, cells (*n* = 3) were dosed with 5 mM stocks of metcamifen (Syngenta, Bracknell, UK) or benoxacor (Syngenta) prepared in dimethyl sulfoxide (DMSO) at a final concentration of 5 μM with DMSO only used as the control. Cells were harvested at timed intervals by vacuum filtration and analyzed immediately.

For whole plant studies, maize seeds (var. Coxximo) were germinated on wet paper and after 3 days were placed in 0.8 mg mL^–1^ (50%) Hoagland’s No. 2 Basal Salt Mixture (pH 5.0) (Sigma-Aldrich) in sealed glass tubes (Figure S2). Seedings were kept in a growth cabinet at 15 h light, 600 μmol m^–2^ s^–1^ (24 °C)/9 h dark (18 °C) at 70% humidity. After growing for 7 days to the two-leaf stage, plantlets were treated with either 25 μM metcamifen or 25 μM benoxacor, with the equivalent dosing solution of 0.1% (v/v) DMSO acting as a control treatment. After 1 h of treatment, plantlets (*n* = 3) were transferred to fresh nutrient solution and were harvested at 3, 24, 48, 72 h and divided into roots, stems, and leaves (Figure S2).

For studies on the expression of CYPs in *planta* from different maize cultivars, seeds of the field hybrid varieties Garland and Maxxis Duo as well as sweet corn hybrid var. Sundance were obtained from Syngenta. Seeds were germinated on wet paper for 3 days before being transferred to a sealed glass tube containing media and kept in a growth cabinet under the environmental conditions described above. After growing for 10 days, plantlets were collected for analysis of the presence of genes encoding functional CYP81A9 and CYP81A16 by PCR amplification of genomic DNA with specific forward and reverse primers (Table S2). The reactions were run in a three-step program: preincubation at 95 °C for 5 min, amplification for 40 cycles (95 °C for 15 s, 57 °C for 30 s, and 72 °C for 60 s), final extension at 72 °C for 5 min. Amplification products were loaded in 1% (w/v) agarose gel, and products were visualized under UV light.

### Herbicide Metabolism Studies with [^14^C]-Mesotrione

Plantlets pretreated with metcamifen or benoxacor as detailed above were transferred into fresh media containing 25 μM [phenyl-U-^14^C]-mesotrione (4.07 MBq mg^–1^, >90% pure). Plantlets were harvested (*n* = 3) at 4.5, 24, 48, and 72 h. The roots were washed with acetonitrile to remove unabsorbed radioactivity and then separated into root, seed, stem, and leaf tissues. Tissues were flash frozen before being pulverized in liquid nitrogen. Finely ground tissues were extracted once with 2 v/w acetonitrile:water (4:1 v/v) and then with 2 v/w acetonitrile:water (1:1 v/v). Combined solvent extracts (50 μL) were radioassayed by liquid scintillation counting (5 mL Prosafe+, TrisKem International, Bruz, France) and then applied onto TLC silica gel 60 F254 plates (Sigma-Aldrich), after standardizing the dpm applied. TLC plates were developed using chloroform:ethyl acetate:methanol:formic acid (30:20:20:2 (v/v)), with cochromatographing reference metabolites visualized under UV light and radioactive metabolites quantified on a Typhoon FLA 9500 phosphorimager (GE Healthcare, Amersham, UK, Multiguage V2.2 software).

### Next-Generation Sequencing

BMS cells (*n* = 3) were treated for 30, 60, 90, and 240 min with either 5 μM metcamifen, 5 μM benoxacor, or 0.1% (v/v) DMSO as a solvent control. Total RNA was extracted and used to generate high-quality cDNA libraries prior to sequencing on a HiSeq 2000 system as described.^8^ Unigenes (34 958) were sequence aligned to the nuclear, chloroplast, and mitochondrial genomes of maize (nuclear B73 RefGen_v2, Maize GDB, Assembly accession: GCA_000005005.4; organelle (http://ftp.maizesequence.org/release-65/gff3/zea_mays/) after taking into account slight differences in unigene GC content, seen in maize.^17^ Differentially expressed genes were identified as described,^8^ after dividing into groups according to treatment (metcamifen versus control and benoxacor versus control) and timing, being analyzed using the EDASeq and edgeR packages in R.^18−20^ Unigenes were considered differentially expressed when log_2_ fold change ≥ 1, and the false discovery rate (FDR) ≤ 0.05. Gene ontology (GO) enrichment was performed for differentially expressed genes using the topGO package in R Studio. The induction of xenome genes including CYPs, GSTs, and ABCs was analyzed using the gplots package of R studio and plotted using the Venn and heatmap.2 functions. CYPs sequences were aligned to 270 CYP genes identified in maize,^12^ using CLUSTALW and the MEGA 6 program with default parameters and identified using the LG+F+G best-fit model,^21,22^ with tree topology adjusted by FigTree v1.4.4. Sequence data linked to this study are registered with NCBI in the GEO depository as id: 267802.

### Functional Assay of Recombinant CYPs

CYP sequences were optimized for expression in *Saccharomyces cerevisiae* and synthesized as double stranded DNA fragments (gblocks, IDT, Coralville, IA, USA), prior to in-fusion cloning (Clontech, St-Germain-em-Laye, France) into pYES3 vectors (Thermo Fisher Scientific, Loughborough, UK). Plasmids were transformed into WAT11 cell lines of *S. cerevisiae*,^23^ with transformants selected using synthetic dropout medium lacking tryptophan, prior to culturing using the “high-density method”.^24^ Recombinant yeast cultures (1 mL) were treated with herbicides at a final concentration of 25 μM and incubated at 30 °C for 24 h. Following centrifugation (13 000*g*, 5 min), the cell-free supernatant was mixed with methanol (1:1) and analyzed by liquid chromatography coupled to mass spectrometry (LC–MS). All recombinant CYPs were also assayed as yeast microsomal preparations.^24^

### Quantification of Recombinant CYPs

Yeast microsome preparations were adjusted to 10 mg of protein mL^–1^ in 50 mM triethylammonium bicarbonate (TEAB), with batches (100 μg) denatured and reduced with 8 M urea and 50 mM dithiothreitol (DTT), prior to alkylation with iodoacetamide. Following a 10-time dilution with TEAB, samples were digested with trypsin (12 h) at a ratio of 1:50 (enzyme:protein), prior to being quenched with formic acid and the addition of labeled internal standard peptides (1 pmol). Samples were then desalted (Oasis μHLB plates, Waters, Herts, UK), dried, and redissolved in water (100 μL) containing formic acid (0.1% v/v) and acetonitrile (3% v/v). The digests were analyzed on a Nano Acquity UPLC liquid chromatograph coupled to a Xevo TQS mass spectrometer (Waters) operating in positive ionization mode with unit mass resolution (Q1 and Q3), with settings of ion spray voltage = 1700 V, ion source temperature = 80 °C, collision gas flow = 0.15 mL min^–1^, nebulizer gas flow = 7.0 bar, and cone voltage = 35 V. The tryptic digest (1 μL) was injected onto a C18 Symmetry trapping column (5 μm, 180 μm × 20 mm; Waters) prior to analytical separation on a BEH C18 column (130 A, 1.7 μm × 200 mm; Waters), at 0.3 μL min^–1^ using 0.1% (v/v) formic acid as mobile phase A and 0.1% (v/v) formic acid in acetonitrile as mobile phase B with a 30 min linear gradient from 10 to 40% B.

For each recombinant CYP, one predicted tryptic peptide was selected for quantification using Skyline Software after missed cleavage sites were disallowed.^25^ Predicted peptides were blasted against reference yeast and maize proteomes, with only sequences specific to a given recombinant CYP scouted on the Waters XevoTQS. In each case, reference peptides were synthesized as heavy labeled standards (JPT, Berlin, Germany), using AQUA methodology.^26^ The results were combined to build a final selection reaction monitored (SRM) method from 64 transitions, representing 10 peptides each targeted with a 120 s detection window. LC–MS chromatograms of the tryptic digests were integrated with Skyline software, with peptide ratios (light:heavy) derived as the average area ratio per transition with analyses performed as three technical replicates.

### CYP Enzyme Activity Assays

Assays were performed in 50 mM Tris-HCl, pH 7.5, containing 0.2 mM herbicide substrate and 1 mM NAPDH, with microsome preparations (250 μg) incubated for 20 to 120 min at 28 °C. Reactions were terminated by added 1 vol of acetonitrile:hydrochloric acid (99:1 v/v) prior to analysis on an Acquity I-class FTN coupled to a Xevo G2-XS QTOF (Waters). Diluted samples (5 μL) were injected onto a BEH C18 (2.1 × 50 mm) column at a flow rate of 0.5 mL min^–1^ and eluted with a gradient starting at 95% A (0.1% formic acid) and 5% B (acetonitrile, 0.1% formic acid) and rising to 95% B over 2.20 min. Eluent was analyzed using electrospray ionization (ESI) using either positive (capillary 0.7 kV) or negative (capillary 2.0 kV) polarity with a source temp at 120 °C, desolvation temperature at 600 °C, and gas flow at 800 L/h. For MS/MS analysis, initially, a low collision energy was used at 10 V and subsequently ramped to 40 V to induce fragmentation. CYP reaction products were identified using reference herbicide metabolites where available or by reference to published mass spectra (Table S1).

### Proteomics of BMS Cell Cultures

BMS cultures (50 g) were treated for 24 h with 5 μM metcamifen, extracted in 100 mM Tris-HCl, pH 7.5, and 15 mM DTT, and following an initial centrifugation (10 000*g*, 15 min, 4 °C), the membrane fraction was recovered by ultracentrifugation (100 000*g*, 90 min, 4 °C). Microsomal preparations were adjusted to a protein concentration of 1 mg mL^–1^ in 50 mM TEAB prior to 100 μg protein batches being digested with trypsin. Digestion was stopped with formic acid (5 μL), and samples were desalted and dried before being redissolved in water (100 μL) containing formic acid (0.1% v/v) and acetonitrile (3% v/v). The tryptic digest sample (1 μg) was injected onto an Acclaim Pep Map C18 trapping column (100 μm × 2 cm, 5 μm, 100 A; Thermo Scientific) at 15 μL min^–1^ for 2 min. The analytical separation was performed at 0.3 μL min^–1^ on a nano Easy Spray C18 column (50 cm × 75 μm, 2 μm, 100 A; Thermo Scientific), using 0.1% (v/v) aqueous formic acid as mobile phase A and 80% (v/v) acetonitrile 0.1% (v/v) formic acid as mobile phase B with a 60 min linear gradient from 5 to 40% phase B. MS/MS spectra were collected on a Q Exactive + mass spectrometer (Thermo Scientific), using the top −10 method, collecting MS spectra at 70K resolution, 3E6 AGC, with a maximum injection time of 50 ms and HCD MS/MS spectra at 17.5K resolution, 2E5 AGC, with a maximum injection time of 100 ms at 28 normalized collision energy. The precursor masses were scanned from 375 to 1800 *m*/*z*, and a dynamic exclusion was employed with a repeat count of 1 and duration of 45 s.

Data analysis was performed using MaxQuant software (1.6.12.0), with files searched against the B73 maize uniport proteome, supplemented with the sequences of CYP81As of interest. All statistical analyses were performed in Perseus with the MaxQuant “proteinGroups.txt” as the primary input file.^27^ LFQ intensities were converted to a log_2_ scale, with replicates grouped by condition (control or treated). Proteins with quantification values in >70% of the samples were kept for statistical analysis, with ANOVA significance testing performed on log-transformed intensities with parameter settings of *s*_0_ = 0.1 and FDR = 0.05.

### RNA Isolation and Quantitative Real-Time PCR (qRT-PCR)

RNA was extracted from frozen plant material (100 mg) using Qiagen RNeasy mini kits, and cDNA was synthesized followed manufacture protocol (Tetro cDNA Synthesis Kit, Meridian BioSciences, London, UK). qRT-PCR was carried out on a Roche LightCycle 96 Real-Time PCR System (Roche Diagnostics Ltd., Burgess Hill, UK). Each reaction (15 μL) consisted of Fast SYBR Green Master Mix (Thermo Fisher Scientific), 6.25 ng of cDNA, and 0.4 μM of specific forward and reverse primers (Table S2). The reactions were run in a three-step program including melting curve analysis; preincubation at 95 °C for 10 min, amplification for 45 cycles (95 °C for 10 s, 60 °C for 10 s, and 72 °C for 20 s; and melting analysis from 65 to 97 °C). Melting curve analysis was used to verify a specific product in each reaction. All reactions were performed with three biological replicates (*n* = 3). Absolute quantification of selected CYPs was determined from the standard curve prepared from serial dilution (10^–1^ −10^–8^) of known quantities (1 ng/reaction) of synthesis double strand DNA fragment (gblock, IDT, Coralville, IA, USA) specific for each CYP.^28^ The mean absolute quantity of transcripts ng^–1^ of total mRNA (mean ± SD) were also calculated.

## Supporting Information Available

**Supplementary Methods**. **Supplemental Tables**: **Table S1** LC–MS analysis of maize herbicides and CYP-derived metabolites. **Table S2** Primer-specific sequences of CYP450s in maize used in real-time qPCR analysis. **Table S3** Changes in global gene expression in maize cell cultures treated with either metcamifen or benoxacor as grouped by their gene ontology. **Table S4** The CYP transcripts identified by RNaseq analysis following treatment of BMS cell cultures with metcamifen or benoxacor as compared to an exposure to carrier solvent. **Table S5** Conversion of bentazon to hydroxybentazon in yeast cultures expressing recombinant maize CYPs. **Table S6** Typical expression levels of members of the CYP81A subfamily expressed as recombinant proteins in yeast microsomes. **Table S7** CYP activities toward the herbicides bentazon, mesotrione, and nicosulfuron in microsomes prepared from BMS cell cultures treated with or without 5 μM metcamifen. **Table S8** Proteomic analysis of polypeptides identified in microsomes prepared from BMS cells treated with 5 μM metcamifen. **Supplemental Figures**: **Figure S1** The herbicides used as CYP substrates in this study. **Figure S2** Schematic of maize seedling treatment with [^14^C]-mesotrione accompanied by safener treatment (benoxacor and metcamifen). **Figure S3** An optimization of *in vitro* assay conditions using microsomal fractions prepared from yeast expressing recombinant CYP81A9. **Figure S4** Phylogenetic tree of safener-inducible CYPs from maize cell suspension cultures. **Figure S5** Amino acid sequence alignment of CYP81A9 and CYP81A16. **Figure S6** Effect of substrate concentration on CYP81A activity. **Figure S7** Structural models of maize CYP450s. **Figure S8** Amplification products of CYP81A9 and CYP81A16 from genomic DNA extracted from seedlings of hybrid cultivars of field maize and sweet corn (PDF)
