# Supplementary material for: Characterization of Cytochrome P450s with Key Roles in Determining Herbicide Selectivity in Maize
Source: ACS Omega. 2022 May 11;7(20):17416–31. doi: 10.1021/acsomega.2c01705 (PMC9134415; doi:10.1021/acsomega.2c01705)
Supplement: Supplementary file 1 — ao2c01705_si_001.pdf [file ao2c01705_si_001.pdf]

Supporting information for

Characterisation of cytochrome P450s with key roles in determining herbicide selectivity in maize

Melissa Brazier-Hicks[1,2], Sara Franco-Ortega[1,3], Philip Watson[1,4], Blandine Rougemont[2], Jonathan Cohn[5], Richard Dale[2], Tim R. Hawkes[2], Alina Goldberg-Cavalleri[1], Nawaporn Onkokesung[1] & Robert Edwards[1]*

[1] School of Natural and Environmental Sciences, Newcastle University, Newcastle upon Tyne, NE1 7RU, UK

[2] Syngenta, Jealott's Hill, Bracknell, Berkshire, RG42 6EY, UK

[3]Current Address, Department of Biology, University of York, York YO10 5DD.

[4]Current Address, Battelle Crop Protection Solutions, Chelmsford Business Park, Essex, CM2 5LB, UK

[5] Syngenta Crop Protection, LLC, 9 Davis Drive, Research Triangle Park, NC 27709-2257, USA

Supplemental method

Phylogenetic Analysis of CYP81s

Alignment of maize CYP81 sequences and homologs from other species, generated within SEAVIEW software version4 .6.4 (Gouy et al. 2010) using Muscle (Edgar 2004) and subsequent trimmed with trimAI software v.1.3 using the settings 'gappyout' (Capella-Gutiérrez et al. 2009) accessed through the webserver Phylemon 2 (Sánchez et al. 2011). The trimmed alignment was used to infer maximum likelihood phylogenies with IQ-TREE software using the online server (Trifinopoulos et al. 2016). The automatic model selection mode was used (Bayesian information Criterion selecting the model JTT+F+G4) and branch support values calculated by Ultrafast bootstrap approximation. The tree was edited with iTOL tool (https://itol.embl.de/), as described (Letunic and Bork 2021).

Phylogenetic analysis was conducted based on 13 *ZmCYP81*s sequences aligned to a selection of homologues from crops and weeds that were previously shown to be able to metabolize herbicides, their expression are modulated by safeners or their expression are enhanced in NTSR biotypes (Tétard-Jones *et al*., 2018, Franco Ortega *et al*., 2021, Pan *et al*., 2006; Iwakami, Uchino *et al*., 2014; Iwakami, Endo *et al*, 2014; Iwakami *et al*., 2019, Duhoux and Délye, 2013; Han *et al* 2020). ZmCYP81A2, ZmCYP81A3, ZmCYP81A9, and ZmCYP81A36 formed of a monophyletic group with high bootstrap support (96) with EpCYP81A12 and EpCYP81A21, a set of proteins that metabolize five chemically unrelated groups of herbicides (Iwakami et al., 2014; Iwakami et al., 2019). Among the four proteins from maize, ZmCYP81A9 is the closest related to EpCYP81A12 and EpCYP81A21.

Supplemental Tables

Table S1 LC-MS analysis of maize herbicides and CYP-derived metabolites. Mass spectra of predicted reaction products of CYP-derived biotransformations of herbicides used in this study along with observed HPLC retention times.

Herbicide		Oxidation location	RT (min)	Parent ion (actual mass)	Mass accuracy (ppm)	Product ion (actual mass)
Mesotrione	Substrate	-	1.32	338.0334 [M-H]-	-0.3	291.0323
	Product	Cyclohexanedione ring	1.05	354.0276 [M-H]-	-2.3	307.0280
Sulcotrione	Substrate	-	1.34	327.0094 [M-H]-	-0.6	291.0308
	Product	Cyclohexanedione ring	1.08	343.0046 [M-H]-	-0.8	307.0258
Tembotrione	Substrate	-	1.67	439.0230 [M-H]-	1.4	403.0453
	Product	Cyclohexanedione ring	1.45	455.0176 [M-H]-	0.7	419.0392
CSCC152531	Substrate	-	1.69	385.0513 [M-H]-	-0.5	349.0730
	Product	nd	1.46	401.0462 [M-H]-	-1.2	365.0685
CSAA464664	Substrate	-	1.78	399.0669 [M-H]-	1.0	363.0894
	Product	nd	1.57	415.0618 [M-H]-	-3.1	379.0831
Topramezone	Substrate	-	0.97	362.0811 [M-H]-	-0.6	334.0483
Nicosulfuron	Substrate	-	1.20	411.1087 [M+H]+	-3.4	182.0564
	Product	Pyrimidine ring	0.93	427.1042 [M+H]+	1.4	198.0512
Rimsulfuron	Substrate	-	1.38	430.0491 [M-H]-	-1.1	154.0612
	Product	Pyrimidine ring	1.09	446.0440 [M-H]-	-1.1	170.0518
Foramsulfuron	Substrate	-	1.22	475.1018 [M+Na]+	1.3	182.0585
	Product	Pyrimidine ring	0.96	491.0969 [M+Na]+	1.6	198.0517
Chlorimuron-ethyl	Substrate	-	1.68	415.0479 [M+H]+	0.7	186.0078
	Product	Pyrimidine ring	1.41	431.0437 [M+H]+	2.3	202.0007
Primisulfuron-methyl	Substrate	-	1.71	491.0261[M+Na]+	-0.6	135.0503
	Product	Pyrimidine ring	1.63	485.0390 [M+H]+	0.0	254.0185
Triasulfuron	Substrate	-	1.37	402.0639 [M+H]+	1.7	167.066
	Product	Phenyl ring	1.26	418.0586 [M+H]+	-0.5	167.0549
Flumetsulam	Substrate	-	1.11	348.0337 [M+Na]+	1.7	129.0405
	Product	Difluorophenyl ring	0.97	364.0295 [M+Na]+	0.8	145.0329
Imazethapyr	Substrate	-	1.50	290.1505 [M+H]+	3.8	177.0672
	Product	nd	0.90	306.1454 [M+H]+	0.0	193.0607
Imazomox	Substrate	-	1.00	306.1454 [M+H]+	2.3	193.0624
Imazaquin	Substrate	-	1.26	312.1348 [M+H]+	1.6	199.0525
Chlorotoluron	Substrate	-	1.41	213.0795 [M+H]+	0.0	140.0268
Isoproturon	Substrate	-	1.47	207.1497 [M+H]+	-1.0	72.0292
Linuron	Substrate	-	1.66	249.0198 [M+H]+	-2.0	182.0247
Atrazine	Substrate	-	1.40	216.6912 [M+H]+	4.1	174.0556
Sethoxydim	Substrate	-	2.19	328.1946 [M+H]+	1.5	282.1537
Bentazon	Substrate		1.41	239.0495 [M-H]-	2.1	132.0305
	Product 1	6-position on carbocyclic ring	1.20	255.0428 [M-H]-	-4.7	148.0254
	Product 2	8-position on carbocyclic ring	1.28	255.0430 [M-H]-	-3.9	148.0252

nd not determined

Table S2 Primer specific sequences of CYP450s in maize used in genomic DNA amplification and real-time qPCR analysis

Type	Gene	Sense (5'-3')	Antisense (5'-3')
**gDNA amplification**	CYP81A9	CGGTTGATGGAGCTCAGGTT	CAGACGATGCCAGCAATTCG
**gDNA amplification**	CYP81A16	TGAAGGGTGTATGACTAT	CCTATTCCATACACTCGGCGA
**qRT-PCR**	CYP81A1	GAGCCTGATGTCTACACGGA	CATTGCCCATTCCAGAGTCG
**qRT-PCR**	CYP81A2	CTGTGCGCCATCTTCTTCTG	GCACGAGGGTATTACGCACT
**qRT-PCR**	CYP81A3	CGGTTGATGGAGCTGAGGTT	AGCGAGAACCTAGGATGTGC
**qRT-PCR**	CYP81A4	CTATGCTTGGCGTCCTCAAG	CGGCTACACTCCAGGAAGAA
**qRT-PCR**	CYP81A9	GGTGTATGACGTAGCTTCCG	CCATTCCATACACTCGGCGA
**qRT-PCR**	CYP81A16	TGAAGGGTGTATGACTAT	CCTATTCCATACACTCGGCGA
**qRT-PCR**	CYP81A17	TGTGTACAACTCGTGAGGCT	CCTGAACATGCTCACGAGTG
**qRT-PCR**	CYP81A36	TGCGTCATGTTCTTCAGCAG	CGAACCATACTGTTACGCGG
**qRT-PCR**	ZmACT	CACTGTGCCCATTTATGAAGGA	CCCGACCAGCAAGATCCA
**qRT-PCR**	DPP9	GAAGGGCCTTGCAAAACCTG	ACCATCCCATGCTGTTACCG

Table S3. Changes in global gene expression in maize cell cultures treated with either metcamifen, or benoxacor, as grouped by their gene ontology (GO) enrichment relating to biological processes (BP), molecular functions (MF) and cellular components (CC) respectively at different treatment times. The table includes the number of annotated unigenes allocated to each GO term in each contrast as significant, expected genes as defined by p-value (Fisher test).

Treatment and time	GO. ID	GO ID	GO Annotation	Annotated	Significant	Expected	P-value
Benoxacor vs Control 30 min. Upregulated	MF	GO:0003950	NAD+ ADP-ribosyltransferase activity	5	1	0.06	0.057
		GO:0008430	selenium binding	5	1	0.06	0.057
Benoxacor vs Control 90 min. Upregulated	CC	GO:0046658	anchored component of plasma membrane	11	2	0.11	0.0036
Benoxacor vs Control 240 min. Upregulated	BP	GO:0055114	oxidation-reduction process	10	1	0.05	0.049
	MF	GO:0019904	protein domain specific binding	15	2	0.17	0.011
	MF	GO:0008430	selenium binding	5	1	0.06	0.054
	CC	GO:0046658	anchored component of plasma membrane	11	2	0.15	0.007
Benoxacor vs Control 240 min. Downregulated	MF	GO:0016853	isomerase activity	16	1	0.04	0.038
Metcamifen vs Control 30 min. Upregulated	BP	GO:0070887	cellular response to chemical stimulus	9	1	0.04	0.044
Metcamifen vs Control 90 min. Upregulated	MF	GO:0003950	NAD+ ADP-ribosyltransferase activity	5	1	0.05	0.052
	CC	GO:0009507	Chloroplast	37	2	0.25	0.015
Metcamifen vs Control 240 min. Upregulated	MF	GO:0003779	actin binding	11	2	0.19	0.015
	MF	GO:0016740	transferase activity	109	6	1.9	0.023
	MF	GO:0019904	protein domain specific binding	15	2	0.26	0.027
	CC	GO:0009507	Chloroplast	37	2	0.25	0.015
Metcamifen vs Control 240 min. Downregulated	BP	GO:0051301	cell division	12	1	0.03	0.03
	MF	GO:0005516	calmodulin binding	12	1	0.03	0.029
	MF	GO:0016853	isomerase activity	16	1	0.04	0.038

Table S4 The CYP transcripts identified by RNAseq analysis following treatment of BMS cell cultures with metcamifen, or benoxacor, as compared to an exposure to carrier solvent (0.1% (v/v) DMSO). CYPs highlighted in grey have a false discovery rate (FDR) of ≥ 0.05 and figures in bold type have an FDR of ≤0.05, being induced more than 2-fold.

			Induction (BMS)					
			Control vs Metcamifen			Control vs Benoxacor		
Clan	Gene	Annotation	30 min	90 min	240 min	30 min	90 min	240 min
71	CYP71S3	GRMZM2G135387	1.02	1.64	**2.57**	1.12	1.65	1.73
	CYP81A2	GRMZM2G069722	1.85	**4.13**	**6.73**	**2.2**	**4.35**	**4.89**
	CYP81A3	GRMZM2G312069	**3.07**	**4.66**	**3.24**	**4.85**	**5.27**	**3.25**
	CYP81A4	GRMZM2G039278	1.47	**7.73**	**18.8**	1.47	**14.92**	**19.54**
	CYP81A9	GRMZM2G090432	**4.22**	**8.01**	**4.06**	**4.22**	**4.9**	**2.73**
	CYP81A36	GRMZM2G114988	**887.67**	**6.21**	**1927.67**	**521**	**5.38**	**1004.33**
	CYP89B19	GRMZM2G181236	**3.76**	**6.11**	**3.57**	**7.9**	**6.34**	**2.57**
72	CYP72A5	GRMZM2G129860	**2.16**	**4.69**	**3.18**	**10.03**	**18.33**	**3.13**
	CYP72A16	GRMZM2G056247	1.09	1.23	**2.03**	1.17	1.42	**2.04**
	CYP72A124	GRMZM2G147752	**2.05**	**2.51**	**6.76**	**4.47**	**5.4**	**8.63**
	CYP72A354	GRMZM2G014395	1.78	**3.35**	**3.71**	**2.26**	**3.33**	**2.78**
	CYP709C14	GRMZM2G075244	**5.57**	**8.06**	**5.85**	**6.96**	**6.41**	**3.09**
	CYP709C21	GRMZM2G075461	**5.06**	**6.81**	**4.76**	**6.29**	**5.82**	**2.67**
	CYP709C24	GRMZM2G106468	1.26	1.66	**2.19**	1.43	1.97	1.85
74	CYP74A18	GRMZM2G072653	1.08	1.47	1.48	1.83	**2.29**	1.49
	CYP74A38	GRMZM2G376661	1.06	1.33	1.41	1.74	**2.04**	1.39
86	CYP704A107	GRMZM2G124972	1.48	**4.96**	**5.75**	**4.58**	**9.47**	**3.9**
	CYP704A108	GRMZM2G066441	1.02	1.54	**2.62**	0.99	1.6	1.89

Table S5 Conversion of bentazon to hydroxybentazon in yeast cultures expressing recombinant maize CYPs as determined as % conversion of the parent to metabolite as recovered in the liquid medium.

Name	Conversion (%)
Empty vector	0
CYP71S3	0
CYP81A1*	18
CYP81A2	52
CYP81A3	0
CYP81A4	13
CYP81A9	20
CYP81A16	20
CYP81A17*	0
CYP81A36	0
CYP89B19	1
CYP72A5	1
CYP72A16	0
CYP72A124	4
CYP72A354	0
CYP709C14	0
CYP709C21	0
CYP709C24	0
CYP74A38	0
CYP704A107	0
CYP704A108	0

*not induced by metcamifen

Table S6 Typical expression levels of members of the CYP81A subfamily expressed as recombinant proteins in yeast microsomes as quantified from a component defined tryptic peptide and normalised to the total protein present in the extracts.

rCYP	Concentration of rCYP in yeast microsomes (pmol rCYP mg^{-1} microsomal protein)	Peptide standard sequences
CYP81A1	Nq	LPYLHCIISETLR
CYP81A2	49.6	LLAAEDVPR
CYP81A3	45.5	LITADDVPR
CYP81A4	67.9	LLGADDLPR
CYP81A9	40.0	DPAVWEDPDR
CYP81A16	10.0	DPAVWDDPDR
CYP81A17	3.8	LLNAGDLPR
CYP81A36	1.7	GTLLFVNAYAIH R

nq not quantified for technical reasons

Table S7 CYP activities toward the herbicides bentazon, mesotrione and nicosulfuron in microsomes prepared from BMS cell cultures treated with or without 5 µM metcamifen

		Specific activity (fkat mg^{-1})	
Substrate	Product	Control	Metcamifen
Nicosulfuron	nc	**0.40** ± 0.16	**1.36** ± 0.32
Mesotrione	4-OH	**0.05** ± 0.01	**0.11** ± 0.00
Bentazon	6-OH	**1.51** ± 0.29	**4.13** ± 0.40
	8-OH	**0.10** ± 0.02	**0.28** ± 0.04

nc not confirmed

Table S8 Proteomic analysis of polypeptides identified in microsomes prepared from BMS cells treated with 5 μM metcamifen.

Protein name	Protein IDs	Fold difference	-Log(P-value)	Unique peptides
CYP81A9 - CYP81A16	B6SSF2	2.26	11.052	10
Mitochondrial phosphate carrier protein 2	A0A1D6I5Q9	1.75	2.621	4
Safener induced1 (In2-1)	B4FT46	1.70	2.580	3
Multidrug resistance-associated protein3	A0A1D6NUZ	1.69	3.424	3
ADP,ATP carrier protein 1	B4G178	1.69	2.596	2
Peptidase family M48 containing protein	B6T55	1.68	2.890	5
ABC transporter G family member 40	A0A1D6I2A0	1.66	3.216	3
CYP81A3	B4G1A3	1.64	9.523	15
ABC transporter B family member 9	A0A1D6NEN5	1.60	9.020	19
Ribonuclease P	C0PAC6	1.58	6.380	2
Polyketide synthesis homolog	A0A1D6DYL8	1.58	4.842	4
Mitochondrial prohibitin complex protein 2	Q9M586	1.57	4.938	5
Polyadenylate-binding protein	A0A1D6E6L0	1.57	5.192	2
Hydrogen-transporting ATP synthase	B6SIP2|	1.48	4.849	2
Phospholipid scramblase	B6TAD3	1.48	5.154	2
Gamma carbonic anhydrase 2 mitochondrial	C0PB60	1.47	2.936	7
Polyadenylate-binding protein	A0A1D6J9Q7	1.46	4.633	4
Inorganic phosphate transporter 3	Q49B44	0.68	4.296	6
Peroxidase	C0HIT1	0.68	8.441	2
Putative sucrose-phosphate synthase	A0A1D6PRE0	0.67	3.346	11
Beta-glucosidase 44	B4G004	0.66	5.629	10
AMP-dependent synthetase and ligase	A0A1D6MDN1	0.66	3.796	8
60S acidic ribosomal protein P1	B4FUB4	0.66	2.536	3
elT/selW/selH selenoprotein domain	K7TN39	0.65	1.943	7
Eukaryotic aspartyl protease family protein	C0PM7	0.63	3.155	3
Gibberellin receptor	B6UC04	0.63	8.266	15
Peroxidase	A0A1D6DVJ2	0.60	7.799	4
40S ribosomal protein S16	B6TG61	0.58	3.774	2
Ferritin	A0A1D6QG81	0.56	0.698	3
Guanine nucleotide-binding protein	B4FKM1	0.55	6.068	5
Guanine nucleotide-binding	B6SJ21	0.53	8.443	6

Supplemental Figures

Triketones (HPPD-inhibitors)

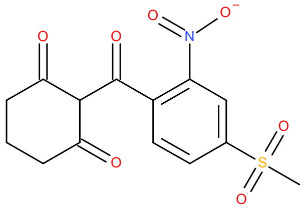

Mesotrione

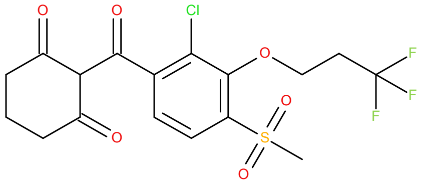

Tembotrione

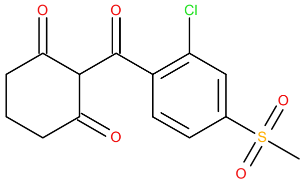

Sulcotrione

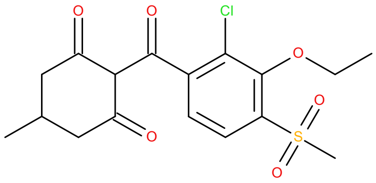

CSCC152531

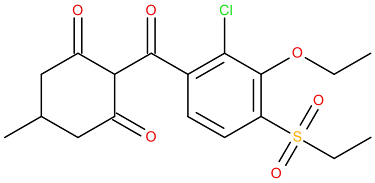

CSAA464664

Benzoylpyrazole (HPPD-inhibitor)

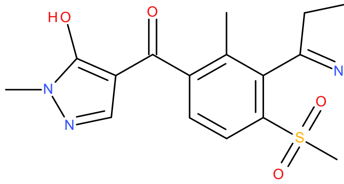

Topramezone

Pyrimidinylsulfonylureas (ALS-inhibitors)

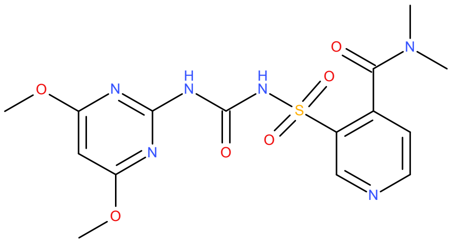

Nicosulfuron

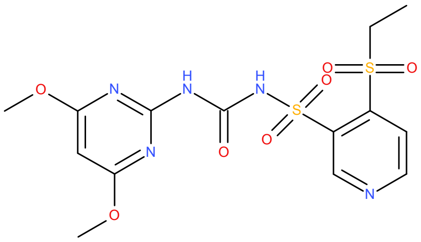

Rimsulfuron

Pyrimidinylsulfonylureas (ALS-inhibitors)

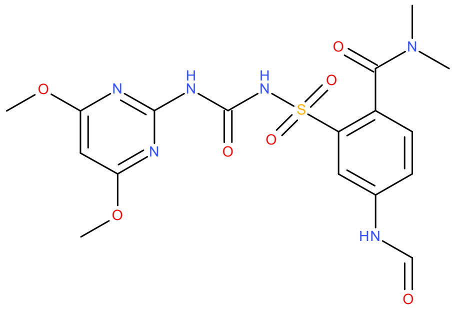

Foramsulfuron

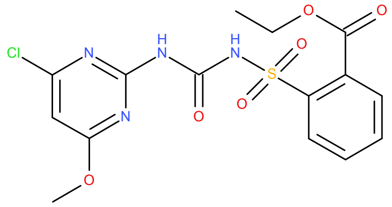

Chlorimuron-ethyl

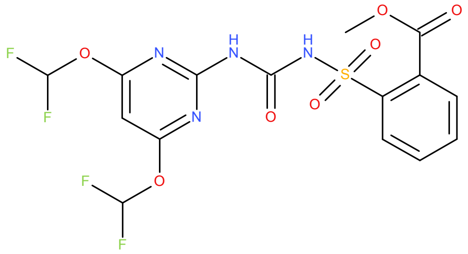

Primisulfuron-methyl

Triazinylsulfonylureas (ALS-inhibitors)

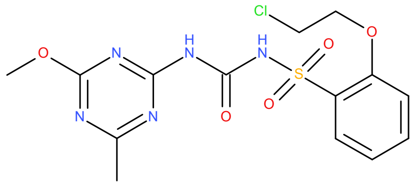

Triasulfuron

Sulfonanilide (ALS-inhibitors)

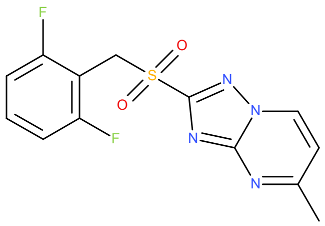

Flumetsulam

Imidazolinone (ALS-inhibitor)

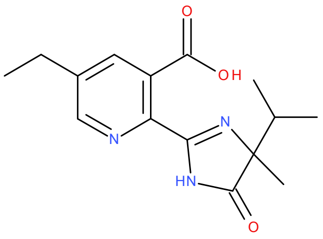

Imazethapyr

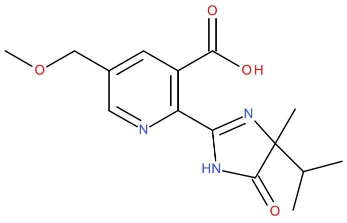

Imazamox

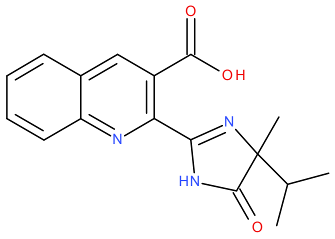

Imazaquin

Phenylureas (PSII-inhibitors)

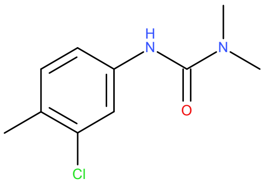

Chlorotoluron

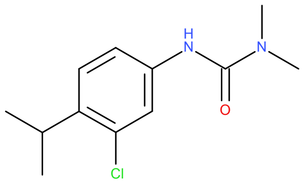

Isoproturon

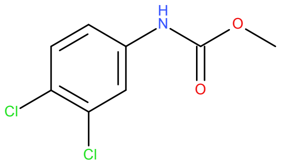

Linuron

Chlorotriazine (PSII-inhibitor)

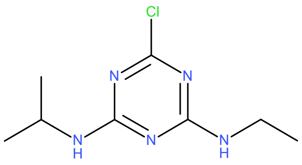

Atrazine

Cyclohexene oxime (ACCase-inhibitor)

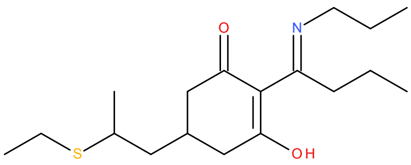

Sethoxydim

Thiadiazine (Growth regulator)

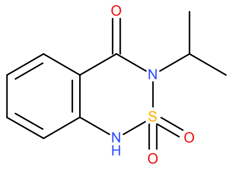

Bentazon

Figure S1. The herbicides used as CYP substrates in this study. The mass ions associated with the parent herbicides and respective oxidised metabolites are presented in Table S1

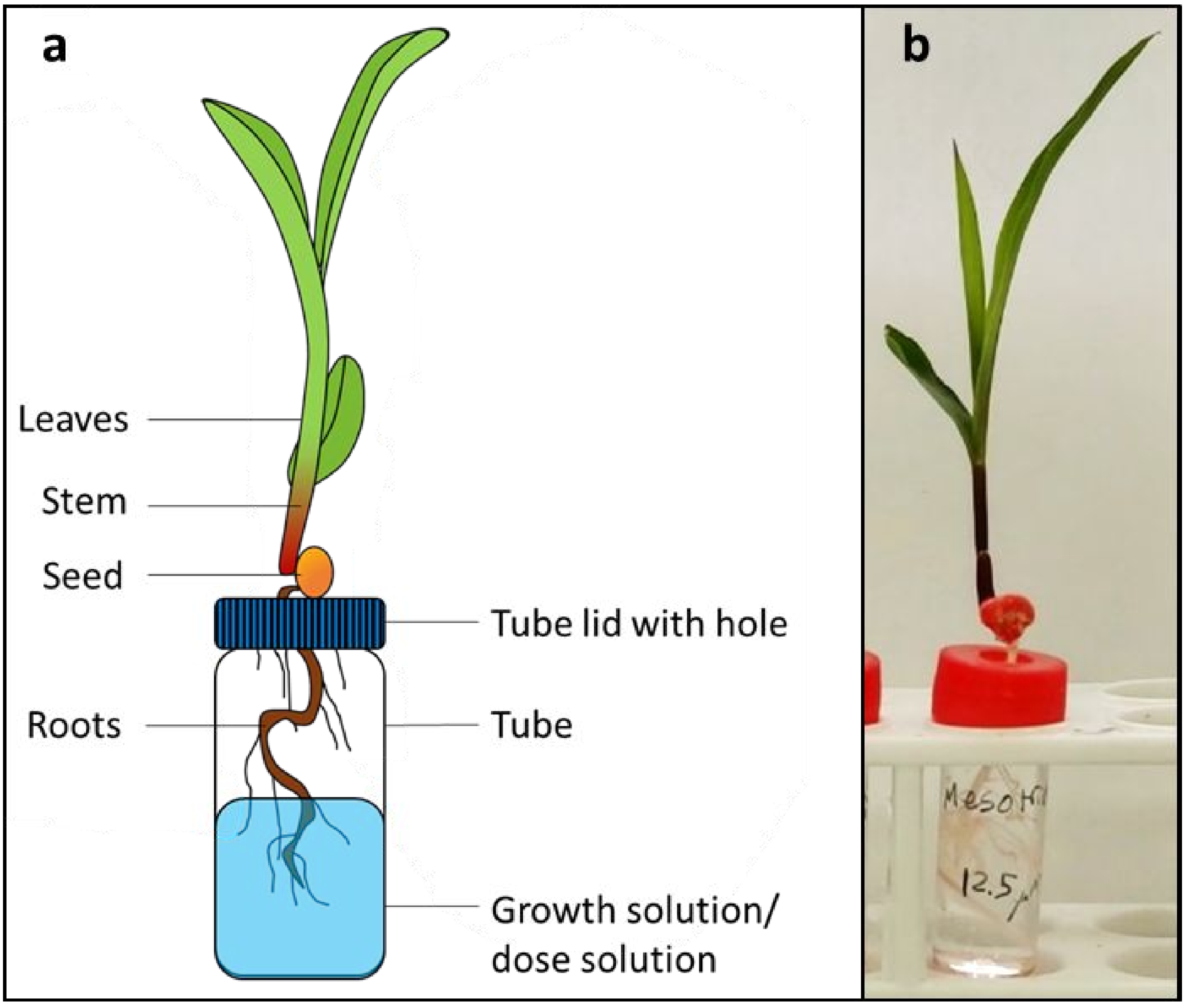

Figure S2. Schematic of maize seedlings treatment with [^{14}C]-mesotrione accompanied by safener treatment (benoxacor and metcamifen). Radiolabelled compound was delivered through the root medium. The separation of tissues for analysis were as shown in schematic a. Photograph courtesy of Philip Watson. Copyright 2019.

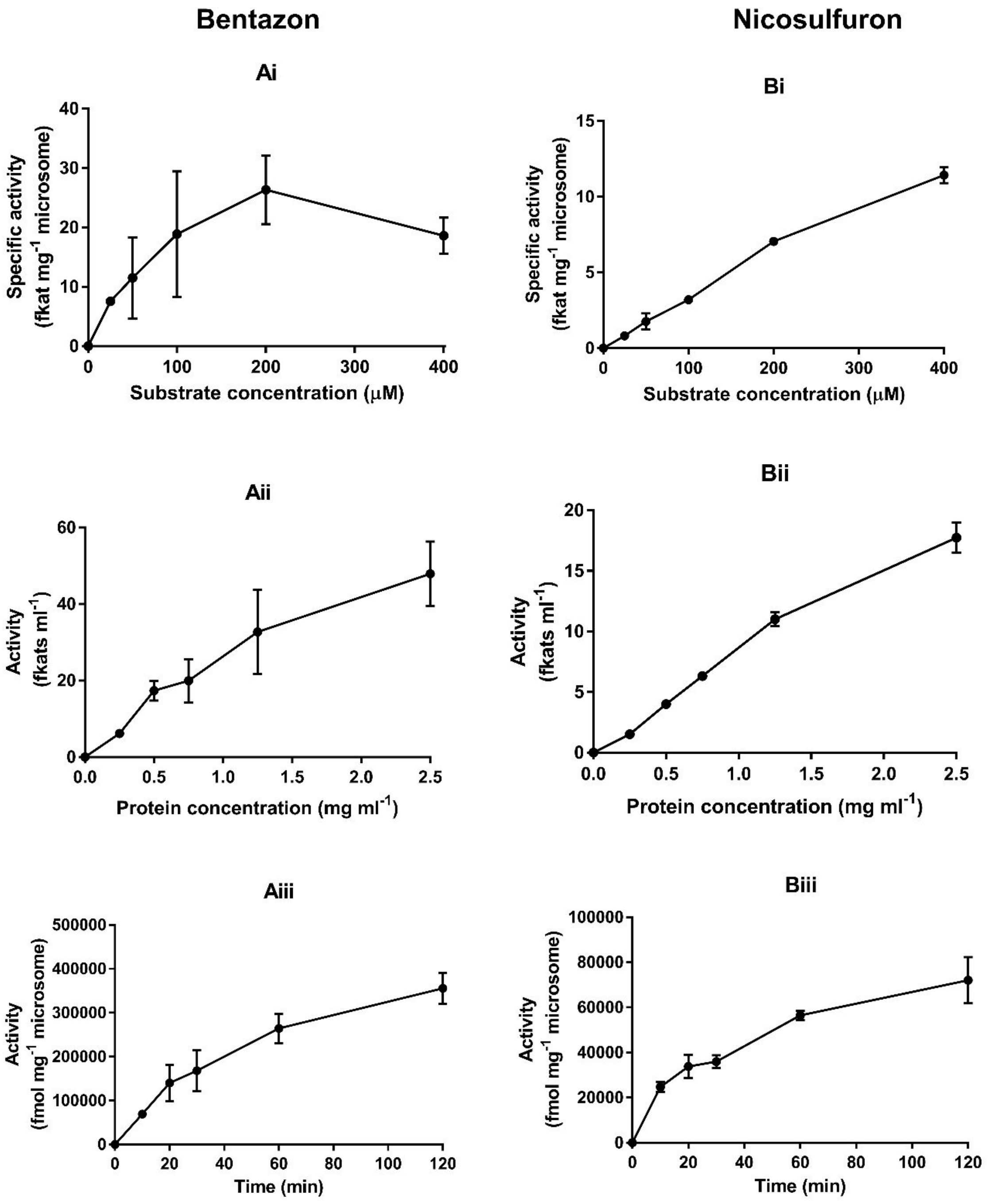

Figure S3. Optimisation of *in vitro* assay conditions using microsomal fractions prepared from yeast expressing recombinant CYP81A9. CYP81A9 activity toward bentazon (A) and nicosulfuron (B) was determined by determining product formation using LC-MS over (i) a range of substrate concentrations, (ii) varied microsomal protein content per reaction and (iii) as a function of reaction time.

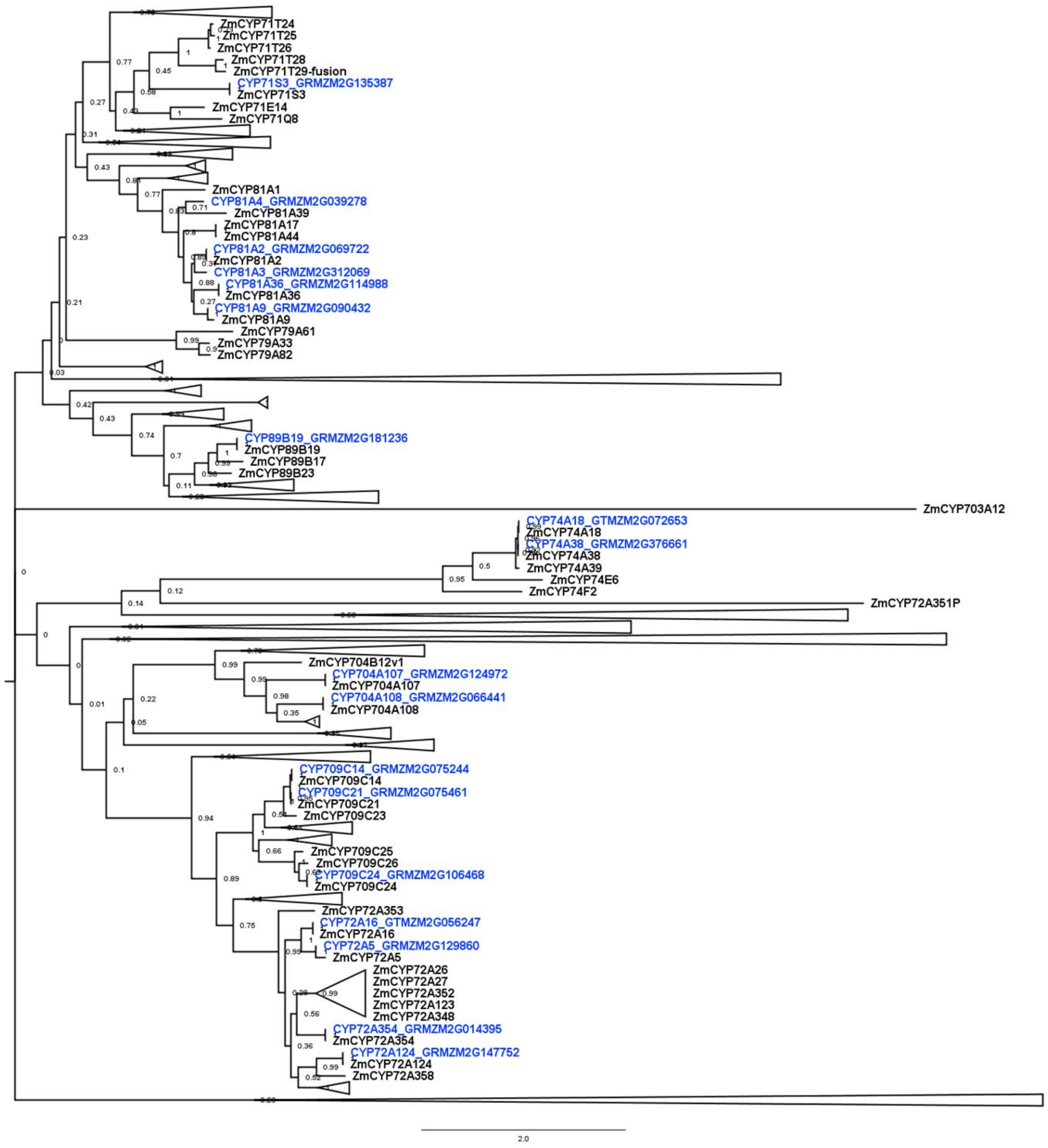

Figure S4 Phylogenetic tree of safener-inducible CYPs from maize cell suspension cultures (in blue) mapped onto the 270 CYPs in maize identified by Li and Wei (2020).

```
              1
ZmCYP81A16   MDKAYIAALSAAALFLLHYL LGRRAGGEG--KTKGSQRRL PPSPPAIPFLGHLHLVKAPF HAALARLAARHGPVFSMRLG TRRAVVVSSPDCARECFTEH
ZmCYP81A9    MDKAYIAALSAAALFLLHYL LGRRAGGEGKAKAKGSRRRL PPSPPAIPFLGHLHLVKAPF HGALARLAARHGPVFSMRLG TRRAVVVSSPDCARECFTEH

            101
ZmCYP81A16   DVNFANRPLFPSMRLASFDG AMLSVSSYGPYWRNLRRVAA VQLLSAHRVACMAPAIEAQV RAMVRRMDRAAAAGGGGAAR VQLKRRLFELSLSVLMETIA
ZmCYP81A9    DVNFANRPLFPSMRLASFDG AMLSVSSYGPYWRNLRRVAA VQLLSAHRVGCMAPAIEAQV RAMVRRMDRAAAAGGGGVAR VQLKRRLFELSLSVLMETIA

            201
ZmCYP81A16   HTKTSRAEADADSDMSPEAH EFKQIVDELVPYIGTANRWD YLPVLRWFDVFGVRNKILDA VGRRDAFLRRLIDGERRRLD AGDDSESKSMIAVLLTLQKS
ZmCYP81A9    HTKTSRAEADADSDMSTEAH EFKQIVDELVPYIGTANRWD YLPVLRWFDVFGVRNKILDA VGRRDAFLGRLIDGERRRLD AGDESESKSMIAVLLTLQKS

            301
ZmCYP81A16   EPEVYTDTVITALCANLFGA GTETTSTTTEWAMSLLLNHR EALKKAQAEIDAAVGTSRLV TADDVPHLTYLQCIVDETLR LHPAAPLLLPHESAADCTVG
ZmCYP81A9    EPEVYTDTVITALCANLFGA GTETTSTTTEWAMSLLLNHR EALKKAQAEIDAAVGTSRLV TADDVPHLTYLQCIVDETLR LHPAAPLLLPHESAADCTVG

            401
ZmCYP81A16   GYDVPRGTMLLVNVHAVHRD PAVWDDPDRFVPERFE--GG KAEGRLLMPFGMGRRKCPGE TLALRTVGLVLGTLLQCFDW DTVDGAQVDMKASGGLTMPR
ZmCYP81A9    GYDVPRGTMLLVNVHAVHRD PAVWEDPDRFVPERFEGAGG KAEGRLLMPFGMGRRKCPGE TLALRTVGLVLATLLQCFDW DTVDGAQVDMKASGGLTMPR

            501
ZmCYP81A16   AVPLEAMCRPRTAMRDVLKR L
ZmCYP81A9    AVPLEAMCRPRTAMRGVLKR L
```

Figure S5 Amino acid sequence alignment of CYP81A9 and CYP81A16

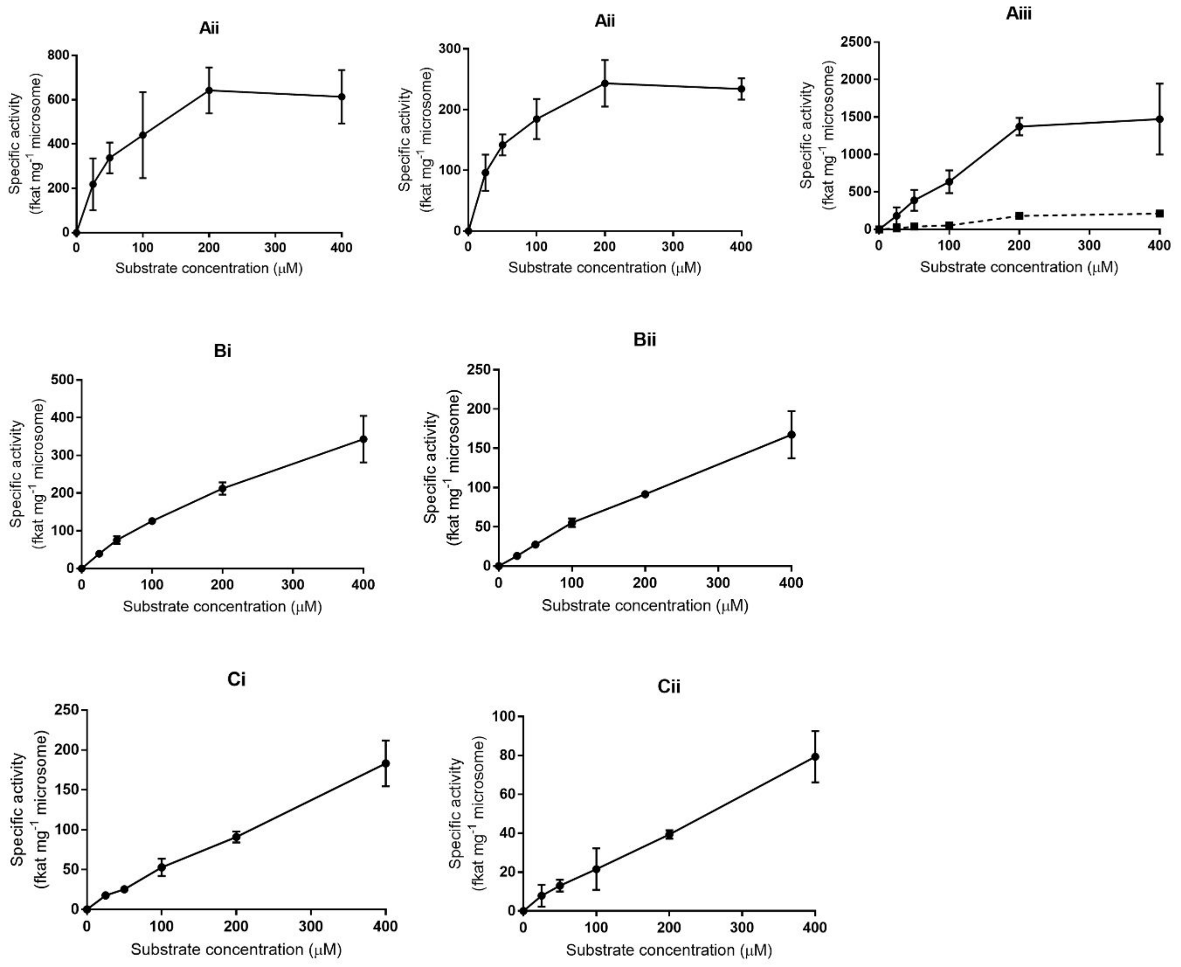

Figure S6 Effect of substrate concentration on CYP81A activity. CYP81A9 (i), CYP81A16 (ii) and CYP81A2 (iii) were assayed for activity toward bentazon (A), nicosulfuron (B) and mesotrione (C).

A

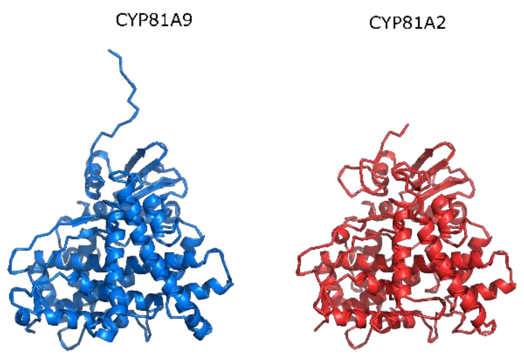

B

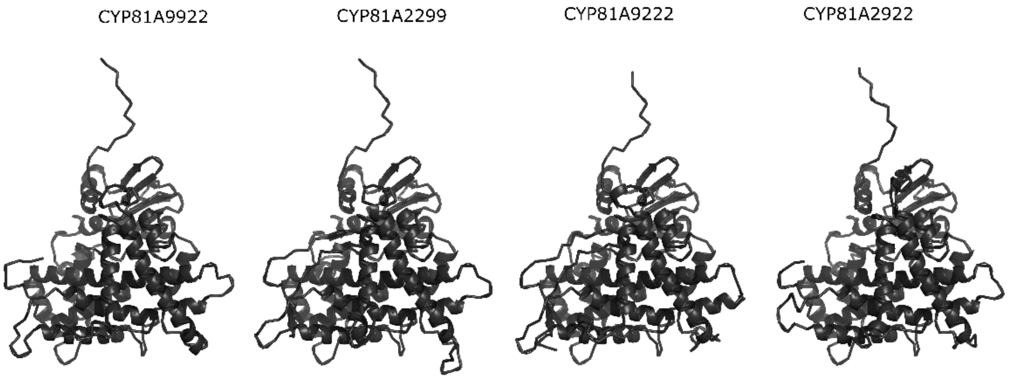

C

```
CYP81A2  ACMSAVVAAEVRAMARRMGRAAAAAPGGAARVQLKRRLFEVSLSVLMETIARTKTSRAEA  61
                 Helix D                              Helix E
CYP81A9  GCMAPAIEAQVRAMVRRMDRAAAAGGGGVARVQLKRRLFELSLSVLMETIAHTKTSRAEA  60

CYP81A2  DADSDMSPEAHEFKQIVDEIVPHLGTANLWDYLPVLRWLDVFGVRNKITAAVGRRDAFLR  121
               Helix F          SRS2         Helix F'     Helix G   SRS3
CYP81A9  DADSDMSTEAHEFKQIVDELVPYIGTANRWDYLPVLRWFDVFGVRNKILDAVGRRDAFLG  120

CYP81A2   RLIDAERRRLDDGGGDSDSDKKS  144
             G continued
CYP81A9   RLIDGERRRLDAG---DESESKS  140
```

Figure S7 Structural models of maize CYP450s. A. CYP81A9 and CYP81A2. B. four chimeras derived from the two maize CYPs using Steroid 17-alpha-hydroxylase/17, 20 lyase (4nkv.3.A) in SwissProt as a template as aligned to the CYP81A9 sequence using PyMOL. C. The alignment of the amino acids sequences in the second quarter of CYP81A9 and CYP81A2. The sequences in boxes predicted to be located in helices, whilst amino acids highlighted in green are putative substrate recognitions sites (SRS).

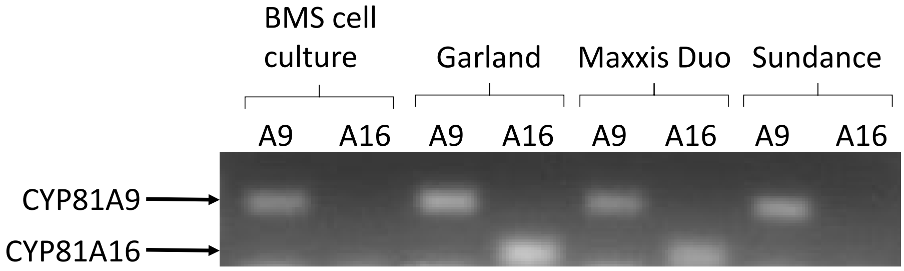

Figure S8 CYP81A9 and CYP81A16 expression in field maize cultivars. Amplification products of CYP81A9 and CYP81A16 from genomic DNA extracted from seedlings of field maize cultivar Garland and Maxxis Duo, and sweetcorn cultivar Sundance. The DNA amplification products were compared with the product amplified from DNA extracted from BMS cell cultures.

REFERENCES

1. Edgar, R. C. MUSCLE: multiple sequence alignment with high accuracy and high throughput. *Nucleic acids Res.* **2004,** *32*, 1792–1797.

2. Gouy, M.; Guindon, S.; Gascuel, O. SeaView version 4: A multiplatform graphical user interface for sequence alignment and phylogenetic tree building. *Mol. Biol. Evol.* **2010**, *27*, 221–224.

3. Capella–Gutiérrez, S.; Silla–Martínez, J. M.; Gabaldón, T. TrimAl: a tool for automated alignment trimming in large–scale phylogenetic analyses. *Bioinformatics* **2009,** *25*,1972–1973.

4. Sánchez, R.; Serra, F.; Tárraga, J.; Medina, I.; Carbonell, J.; Pulido, L.; de Maria, A.; Capella-Gutierrez, S.; Huerta-Cepas, J., Gabaldon, T.; et al. Phylemon 2.0: a suite of web–tools for molecular evolution, phylogenetics, phylogenomics and hypotheses testing. *Nucl. Acids Res.* **2011,** *39*, 470–474.

5. Gouy M.; Guindon S.; Gascuel O. SeaView version 4: a multiplatform graphical user interface for sequence alignment and phylogenetic tree building. *Mol. Biol. Evol.* **2010**, *27* 221–224.

6. Trifinopoulos, J.; Nguyen, L-T.; von Haeseler, A.; Minh, B.Q. W-IQ-TREE: a fast online phylogenetic tool for maximum likelihood analysis, *Nucleic Acids Res*. **2016**, *44*, 232–235.

7. Letunic,I.; Bork P. Interactive Tree Of Life (iTOL) v5: an online tool for phylogenetic tree display and annotation, *Nucleic Acids Res.* **2021**, *49*, 293-296.

8. Iwakami, S.; Endo, M.; Saika, H.; Okuno, J.; Nakamura, N.; Yokoyama, M.; Watanabe, H.; Toki, S.; Uchino, A.; Inamura, T. Cytochrome P450 CYP81A12 and CYP81A21 Are Associated with Resistance to Two Acetolactate Synthase Inhibitors in *Echinochloa phyllopogon*. *Plant Physiol.* **2014**, *165,* 618-629.

9. Iwakami, S.; Uchino, A.; Kataoka, Y.; Shibaike, H.; Watanabe, H.; Inamura. T. Cytochrome P450 genes induced by bispyribac-sodium treatment in a multiple-herbicide-resistant biotype of *Echinochloa phyllopogon. Pest Manag Sci*. **2014**, *70*. 549-58.

10. Franco-Ortega, S.; Goldberg-Cavalleri, A.; Walker, A.; Brazier-Hicks, M.; Onkokesung, N.; Edwards, R. Non-target Site Herbicide Resistance Is Conferred by Two Distinct Mechanisms in Black-Grass (*Alopecurus myosuroides*). *Front Plant Sci*. **2021**, *12*:636652.

11. Tétard-Jones, C.; Sabbadin, F.; Moss, S.; Hull, R.; Neve, P.; Edwards, R. Changes in the proteome of the problem weed blackgrass correlating with multiple-herbicide resistance. *Plant J.* **2018**, *94:*709-720.

12. Duhoux, A.; Délye, C. Reference Genes to Study Herbicide Stress Response in *Lolium* sp.: Up-Regulation of P450 Genes in Plants Resistant to Acetolactate-Synthase Inhibitors. *PLoS ONE* **2013,** *8*:e63576.

13. Pan. G.; Zhang, X.; Liu, K.; Zhang, J.; Wu, X.; Zhu, J.; Tu, J. Map-based cloning of a novel rice cytochrome P450 gene CYP81A6 that confers resistance to two different classes of herbicides. *Plant Mol. Biol.* **2006,** *61,* 933–943

14. Han, H.; Yu, Q.; Beffa, R.; González, S.; Maiwald, F.; Wang, J.; Powles, S.B. Cytochrome P450 CYP81A10v7 in *Lolium rigidum* confers metabolic resistance to herbicides across at least five modes of action. *Plant J.* **2020,** *105,* 79-92.
